# Single-molecule real-time transcript sequencing identified flowering regulatory genes in *Crocus sativus*

**DOI:** 10.1186/s12864-019-6200-5

**Published:** 2019-11-14

**Authors:** Xiaodong Qian, Youping Sun, Guifen Zhou, Yumei Yuan, Jing Li, Huilian Huang, Limin Xu, Liqin Li

**Affiliations:** 1 Huzhou Central Hospital, Huzhou Hospital affiliated with Zhejiang University, Huzhou, 31300 Zhejiang China; 2 Department of Plant, Soil and Climate, Utah State University, Logan, 84322 USA; 3 Department of Chinese Medicine, Zhejiang University of Traditional Chinese Medicine, Hangzhou, 310053 Zhejiang China

**Keywords:** Saffron, Flower, SMRT sequencing, qRT-PCR, Alternative splicing

## Abstract

**Background:**

Saffron crocus (*Crocus sativus*) is a valuable spice with medicinal uses in gynaecopathia and nervous system diseases. Identify flowering regulatory genes plays a vital role in increasing flower numbers, thereby resulting in high saffron yield.

**Results:**

Two full length transcriptome gene sets of flowering and non-flowering saffron crocus were established separately using the single-molecule real-time (SMRT) sequencing method. A total of sixteen SMRT cells generated 22.85 GB data and 75,351 full-length saffron crocus unigenes on the PacBio RS II panel and further obtained 79,028 SSRs, 72,603 lncRNAs and 25,400 alternative splicing (AS) events. Using an Illumina RNA-seq platform, an additional fifteen corms with different flower numbers were sequenced. Many differential expression unigenes (DEGs) were screened separately between flowering and matched non-flowering top buds with cold treatment (1677), flowering top buds of 20 g corms and non-flowering top buds of 6 g corms (1086), and flowering and matched non-flowering lateral buds (267). A total of 62 putative flower-related genes that played important roles in vernalization (VRNs), gibberellins (G3OX, G2OX), photoperiod (PHYB, TEM1, PIF4), autonomous (FCA) and age (SPLs) pathways were identified and a schematic representation of the flowering gene regulatory network in saffron crocus was reported for the first time. After validation by real-time qPCR in 30 samples, two novel genes, PB.20221.2 (*p* = 0.004, *r* = 0.52) and PB.38952.1 (*p* = 0.023, *r* = 0.41), showed significantly higher expression levels in flowering plants. Tissue distribution showed specifically high expression in flower organs and time course expression analysis suggested that the transcripts increasingly accumulated during the flower development period.

**Conclusions:**

Full-length transcriptomes of flowering and non-flowering saffron crocus were obtained using a combined NGS short-read and SMRT long-read sequencing approach. This report is the first to describe the flowering gene regulatory network of saffron crocus and establishes a reference full-length transcriptome for future studies on saffron crocus and other Iridaceae plants.

## Background

*Crocus sativus* L, commonly known as saffron crocus, is prized for purple flowers that are well known for producing spice saffron from the filaments. Spice saffron is the most valuable spice used as a fabric dye and in traditional medicine with special medicinal effects of promoting blood circulation, cooling blood and detoxifying, thereby relieving depression and soothing nerves [1]. As a valuable traditional Chinese medicine, saffron is widely used in China and Europe. Saffron crocus blooms only once a year and unlike most spring-blooming plants, saffron crocus does not blossom until autumn. In China, the daughter corms began to grow at the end of January and matured at the end of May and subsequently, entered a dormant period until mid-August. During the period, the corms were dug out from the soil when the leaves turned yellow and wilted and moved into the door to store. Experiencing the high temperature treatment in summer (ranged from 23 to 27 °C), the buds were broken up from dormancy in the middle of August and the floral primordia began to initiate. When the average room temperature fell to 15–17 °C in mid-autumn, most apical buds were in blossom [2]. Basically, the corms had 1–3 apical buds and 6–10 lateral buds depending on their weight. Each apical bud germinated 1–3 flower primordia while lateral bud usually did not blossom. Occasionally, one or more lateral buds of corms weighing more than 30 g also blossomed. The corms weighing less than 6 g cannot blossom. As soon as all the flowers were picked up indoors, the corms were planted in the soils until the new daughter corms matured in the next May. Planting and harvesting corms as well as collecting red stigmas from flowers, is performed manually. To produce 1 kg of dry saffron, 110,000–170,000 flowers are harvested and 40 h of labour are needed to pick 150,000 flowers. Such labour-intensive cultivation practices make saffron a high expensive crop with prices ranging from $500 to $5000 per pound at wholesale and retail rates [3]. Due to limited natural resources for saffron crocus plants, inefficient cultivation, and low yield, saffron is becoming even moreexpensive and is well known as “red gold” [3]. It is highly important to explore comprehensive genetic information for breeding and improving its biological traits.

Increasing the flower number of saffron crocus is a viable way to produce more saffron to meet the ever-increasing demand in the market [4, 5]. Research has been conducted to investigate the factors that affect floral development including temperature, photoperiod, corm size, and bud position [2, 6]. We can obtain samples of different flowering quantities by controlling these factors artificially. Therefore, *C. sativa* is a good material for studying the development of flowering. Many genes related to plant floral development have been discovered along with the rapid development of technology in molecular biology. For example, long-day conditions can promote *Arabidopsis* flowering through the function of FLOWERING LOCUS T (FT) protein, which is considered to be the main component of “florigen” [7, 8]. The transcription factor Flowering Locus C (FLC) is a key regulator of the vernalization process of *Arabidopsis thaliana*. The transcription factor PIF4 is a major regulator of high temperature-induced flowering [9]. Using the FT gene in *Arabidopsis thaliana* as a reference, Tsaftaris et al. cloned a CENTRORADIALIS/TERMINAL FLOWER1 (CsatCEN/TFL1) like gene [10] and three FT-like genes [11] from the flowers, flower buds, leaves, and corms of saffron crocus, respectively, and further proved that their expression patterns were tissue-specific and depended on the flower developmental stage. Other studies found a serial potential flower-related gene in saffron crocus, for instance, B-class paleo AP3-like genes (CsatAP3-like) [12], AP1-like MADS-box genes [13], B-class floral homeotic genes PISTILLATA/GLOBOSA [14], E-class SEPALLATA3-like MADS-box genes [15], and CsMYB1, a transcription factor belonging to the R2R3 family [16]. Later, NGS-based RNA-seq technology was widely used for gene discovery, which led to the identification and functional characterization of flowering genes in various species. For example, *trehalose 6-phosphate synthase* and *squamosa promoter-binding protein-like* genes promoted the floral induction of apple trees [17]. A series of genes related to the circadian clock are important key regulators for the flower development of *hibiscus* [18]. Using NGS-based RNA-seq technology, Baba et al. [19] and Jain et al. [20] discovered the gene expression of saffron crocus involved in apocarotenoid biosynthesis and further explored the expression profiling of zinc-finger transcription factors [21]. However, the underlying molecular mechanism controlling and/or affecting the number of flowers of saffron crocus has not been determined. The genome has not been fully elucidated to date, even in the whole Iridaceae family, only de novo assembly based short-fragment transcriptome of saffron crocus was provided by Illumina RNA-seq sequencing [19–21].

Recently, the third-generation sequencing platform, SMRT sequencing, developed by PACBIO RS (Pacific Biosciences of California, Menlo Park, CA, USA), was used in transcript sequencing. The sequencing platform is good for long reads with an averaged read length of > 10 kb, and real length can reach 60 kb (http://www.pacb.com/smrt-science/smrt-sequencing/read-lengths/). After correction by next generation sequence (NGS) reads and self-correction via circular-consensus sequence (CCS) reads, the error rate of SMRT sequencing is expected to be 1% [22]. This technology has been applied to access complete transcriptome data of a few plants, including *Carthamus tinctorius* (safflower) [23], *Cassia obtusifolia* (Jue-ming-zi) [24], *Panax ginseng* (Korean ginseng) [25], *Salvia miltiorrhiza* (danshen) [26], *Sorghum bicolor* (sorghum) [27] and *Zea mays* (maize) [28].

Compared with the NGS platform, PacBio Iso-Seq can obtain a collection of high quality full-length transcripts without assembly, which is especially important for species without reference genome sequences. Some transcripts might contain repeat regions, whereas transcripts of different gene isoforms show high sequence similarity. The assemblies of short sequencing reads often encounter complications without reference genome sequences. The problem seems more severe for saffron crocus, because of its relatively larger genome size [29] (greater than 10 Gb) and polyploid characteristics [30] (2n = 3x = 24). Saffron crocus consists mainly of repetitive DNA sequences, such as retrotransposon and satellite DNA [31], resulting in particular challenges for the accuracy of short-read assembly. The PacBio Iso-Seq technology can overcome these difficulties by generating sequence information for the full length sequence as a single sequence read without further assembly.

In this paper, NGS and SMRT sequencing were combined to generate two sets of full-length transcriptomes of flowering and non-flowering saffron crocus. Moreover, differentially expressed full-length transcripts of flowering and non-flowering saffron crocus were identified and characterized.

## Materials and methods

### Plant materials

Saffron crocus plants were cultivated at a research farm at South Tai Lake Agricultural Park, Huzhou (longitude 120.6° E, latitude: 30.52° N, elevation 0 m), using a two-stage cultivation method: corms planted in soil to allow them to grow outdoors and be cultivated indoors without soil [32]. In May 2016, dormant corms were excavated from the field and stored indoors for approximately half a year until flowering.

Two sample pools were set up to establish the PacBio Iso-seq libraries of flowering saffron crocus and non-flowering saffron crocus separately. One sample pool was constructed for the full-length transcript set of flowering saffron crocus, which included 1) top bud tissues, 2) tuber tissues of flowering corms (5–7 mm, ≈20 g) (recently differentiated flower primordia and leaf primordia), 3) pistils, 4) stamens of flowering corms (≈20 g) when colours turned from yellow to red, and 5) leaves of flowering corms (≈20 g) when colours turned from white to green), and 6) purple petals of flowering corms (≈20 g). The other sample pool was constructed for the full-length transcript set of non-flowering saffron crocus, which included 1) top bud tissues, 2) lateral bud tissues, 3) tuber tissues of non-flowering corms (5–7 mm, ≈20 g), 4) leaves of non-flowering corms (≈20 g) when turned from white to green, and 5) top bud tissues of non-flowering corms (5–7 mm, ≈6 g) (Additional file 1: Figure S1).

Meanwhile, an additional five groups of saffron crocus corms were prepared to construct higher-accuracy short-read libraries using an Illumina RNA-seq method. The sample pools included 1) top buds of flowering saffron crocus corms, 2) paired top buds of non-flowering saffron crocus corms (≈20 g) that were split into two parts and cultivated at room temperature (20–25 °C, flowering phenotype) and 10 °C (non-flowering phenotype) for 15 days, 3) lateral buds of flowering saffron crocus corms, 4) paired lateral buds of non-flowering saffron crocus corms (≈30 g), and 5) top buds of non-flowering saffron crocus corms (≈6 g) (Additional file 1: Figure S1). All five bud samples were collected when they were 5–7 mm long. A total of 15 plants, (three plants per group) were harvested to construct 15 Illumina RNA-seq libraries.

All the samples prepared for both PacBio Iso-seq and Illumina RNA-seq sequencing were immediately frozen in liquid nitrogen until RNA was isolated.

### RNA preparation

All tissues were ground in liquid nitrogen and total RNA was extracted using an RNeasy@Plant Mini Kit (Qiagen Corporation, Hilden, Germany) according to the manufacturer’s protocol. The isolated RNA samples were detected using 1% agarose electrophoresis to avoid degradation and genomic DNA contamination. RNA purity (OD 260/280 = 2.0–2.2, A260/A280 = 1.8–2.1) was quantified using a Nanodrop 2000 (Thermo Scientific, Waltham, MA, USA), and the concentration of RNA samples was quantified using a Qubit 2.0 Fluorometer (Thermo Scientific, MA, USA). RIN Integrity Number (RIN) values and 28S/18S (28 s: 18 s > = 1.5, RIN > = 8) were measured using an Agilent 2100 Bioanalyzer (Agilent, Santa Clara, CA, USA).

### PacBio Iso-Seq library preparation and sequencing

**Fig. 1:**
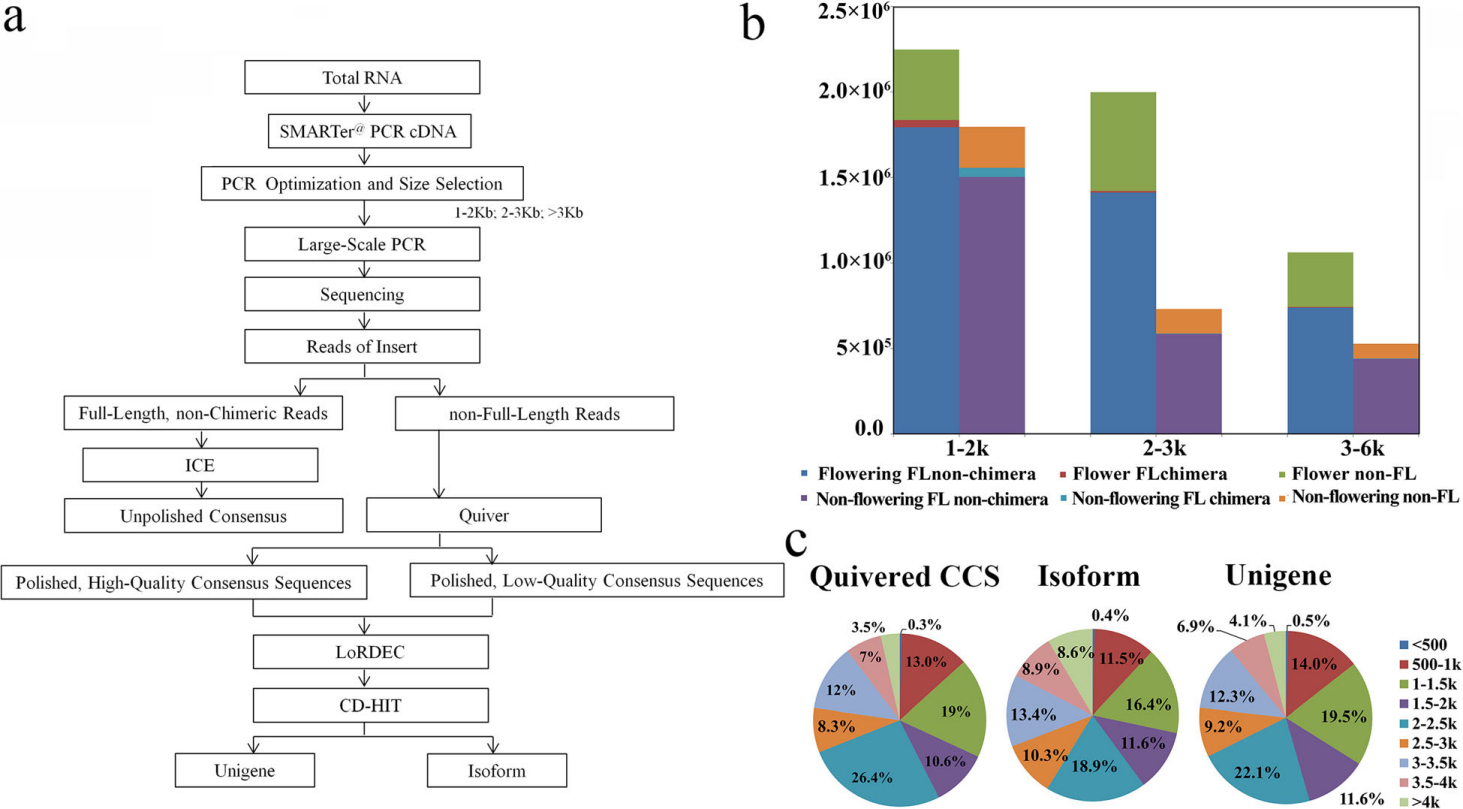
Full-length transcriptome analysis from PacBio Iso-seq. **a**: The workflow for the whole PacBio Iso-seq data processing. **b**: distributions of Full length (FL) non-chimaera, FL chimaera and non-FL chimaera in flowering and non-flowering saffron crocus libraries. **c**: Length distributions of Quivered CCS reads, isoforms and unigenes

PacBio Iso-Seq libraries of flowering and non-flowering saffron crocus were constructed separately. After RNA samples were tested, total RNAs from each set of sample pools (flowering/non-flowering saffron crocus) were mixed and isolated for Poly (A) RNA using a Poly (A) Purist™ MAG Kit (Invitrogen, Carlsbad, CA, USA). Poly (A) RNA was reverse transcribed into cDNA using a SMARTer® PCR cDNA Synthesis Kit (Clontech, Mountain View, CA, USA) with SMARTScribe® MMLV RT enzyme (Takara, Dalian, China). The cDNA products were further amplified with the optimal number of cycles using KAPA HiFi PCR Kits. The PCR products were screened using a BluePippin® Size Selection System (Sage Science, Beverly, MA, USA), and three fractions containing fragments of 1–2, 2–3, and > 3 kb in length were obtained. The sorted fragments of PCR products were amplified again using KAPA HiFi PCR Kits to produce enough DNAs for constructing sequencing libraries. The PCR products were subjected to construct SMRTBell libraries using a SMRTBell Template Prep Kit (Pacific Biosciences, Menlo Park, CA, USA) after fragment ends were repaired and the blunt hairpin adapters at both ends of the DNA fragments were connected. A total of 16 SMRT cells, that is, eight SMRT cells (3 cells for the 1–2 kb library, 3 cells for the 2–3 kb library and 2 cells for the > 3 kb library) run for each sample pool, were analysed using a PacBio RS II platform (Pacific Biosciences, Menlo Park, CA, USA). Figure 1a lists the workflow for the whole PacBio Iso-seq data processing.

### Illumina RNA-seq library preparation, sequencing, and Contigs assembly

Fifteen RNA samples from saffron crocus buds were used for Illumina RNA-seq library construction and sequencing. Total RNA was enriched using Oligo (dT) magnetic beads and randomly broken into short fragments that were further used as a template to synthesize cDNA with random hexamer-primers. The cDNA products were end-repaired, A-tailed, and added with Illumina paired-end adapters. The fragments were selected using AMPure XP beads and PCR amplified to obtain sequencing libraries that were qualified and paired-end sequenced with an Illumina Hiseq 2000 (Illumina, San Diego, CA, USA).

The raw reads of the sequences were obtained by removing adapter reads, reads with length of < 100 bp, and reads with content of ambiguous bases ‘N’ > 5%. De novo assembly of transcriptome sequencing without reference genome, including steps of Inchworm, Chrysalis, and Butterfly with default parameters was conducted using Trinity software.

### Quality control, error correction of PacBio reads and Contigs mapping between corrected PacBio reads and Contigs from RNA-seq

The raw data from the PacBio RS II platform were filtered using SMRTLink software (version 4.0) to obtain Post-Filter Polymerase reads, namely, CCS, when the adaptors, subreads < 50 bp, polymerase reads < 50 bp and accuracy of polymerase reads < 0.75 were deleted. CCS were further self-corrected and filtered with the criterion of full passes > 1 and the predicted consensus accuracy > 0.8 toobtain high-quality reads of inserts (ROIs). ROIs were classified into non-full-length reads and full-length reads (including full-length non-chimeric reads and full-length chimeric reads) based on the presence and location of 3′ primer, 5′ primer and polyA. Full-length non-chimeric reads were corrected by the CEC algorithm and produced Unpolished Consensus Sequences (UCS). The UCS and the remaining ROIs were further corrected using Quiver software to obtain polished high-quality isoforms (accuracy > 0.99) and polished low-quality isoforms.

Subsequently, all Quiver-polished isoforms were mapped to Trinity-assembled contigs from RNA-seq to produce Trinity-corrected Pacbio Isoforms using LoRDEC software [33]. By aligning the Trinity-corrected Pacbio Isoforms to contigs assembled by Trinity with a high level of similarity (> 99% threshold), the longest contigs were assigned to the duplication-removed and corrected long reads (DRCLR). The DRCLR was corrected to remove redundant information using CD-HIT software (version 4.6) and regarded as Unigene.

### Unigene annotation

To predict unigene function, unigenes were searched against five databases, including Cluster of Orthologous Groups of proteins (COG), SwissProt, NCBI non-redundant (NR), Gene Ontology (GO) and Kyoto Encyclopedia of Genes and Genomes (KEGG). Functional annotation of unigenes was obtained from sequence similarity alignment using the BLAST algorithm with a criterion of E-value <1e-10.

### Prediction of coding DNA sequence and protein

All the isoforms were used to predict the coding sequences (CDS) and protein sequences using ANGEL software with *Arabidopsis thaliana* and *Phalaenopsis equestris* (orchid) genomes as the reference genomes. The genome of the Iridaceae family has not been fully elucidated to date [19]. Among all species with known genomes released recently, *Phalaenopsis equestris* has the most homology with saffron crocus [34].

### SSR annotation and long non-coding RNA identification

SSRs (simple sequence repeats) were searched using MISA software (version 1.0) [35]. Long non-coding RNAs (lncRNA) were predicted according to the guiding principles of lncRNAs pipeline (https://bitbucket.org/arrigonialberto/lncrnas-pipeline) with PLEK (an improved k-mer scheme tool) as the core algorithm [36]. PLEK is widely used to discriminate protein-coding mRNAs and non-coding RNAs and has the ability of predict all possible open reading frames (ORFs) and translate the sequences into peptides.

### Alternative splicing analysis and validation

The alternative splicing (AS) events were predicted based on the BLAST alignment of DRCLR to the Trinity-assembled contigs from RNA-seq sequencing using default parameters. AS events were defined when the alignment gaps were longer than 50 bp and were at least 100 bp from the 3′ and 5′ ends [33]. The specific AS presented in only the PacBio Iso-seq library of flowering or non-flowering saffron crocus were screened separately. To validate the accuracy of the AS detected with PacBio Sequencing, RT-PCR of three randomly selected unigenes, PB.174, PB.313 and PB.988,was performed. Total RNA of saffron crocus buds was extracted as described above. The PrimeScript II 1st Strand cDNA Synthesis Kit (TaKaRa, Japan) and SYBR Premix Ex Taq II (TaKaRa, Japan) were used for reverse transcription reaction and PCR assay. Specific primers (Additional file 2:Table S1) of the chosen genes were designed using Primer Premier 5.0 software (Premier, Vancouver, British Columbia, Canada) according to the homologous sequences at the upstream and downstream ends of all the different alternative splicing fragments. The PCR amplification procedure included 98 °C 10 s, 56 °C 30 s, 72 °C 3 min for 30 cycles and then 72 °C extended for 5 mins. PCR products were monitored by 1% agarose gel electrophoresis. Sequencing of the PCR products further confirmed the correctness of the amplification.

### Screening differentially expressed Unigenes and GO and KEGG enrichment analyses

The expression levels of all the unigenes in fifteen samples were assayed based on the Illumina short reads dataset, and reference sequences were the unigene libraries. Relative gene expression levels of each unigene were determined by FPKM (fragments per kilobase of transcript per million mapped reads) and differentially expressed unigenes were screened using DESeq2 R with parameter cutoff *p*-value < 0.05, FDR < 0.01 and fold change ratio > 2.

Differentially expressed unigenes were also employed for the enrichment analyses of GO and KEGG pathway with adjusted *p*-value (q-value) < 0.05 serving as the standard for significantly enriched pathway.

### Validation of differentially expressed Unigenes using real-time qRT-PCR

Twenty (8 flowering and 12 non-flowering) top buds and ten (4 flowering and 6 non-flowering) lateral buds of saffron crocus with various corm weighst and bud lengths were used to validate differentially expressed unigenes using real-time quantitative reverse transcription PCR (qRT-PCR). Eleven differentially expressed unigenes between flowering and non-flowering samples were selected for validating key flower unigenes. All buds were ground in liquid nitrogen, and total RNA was prepared using an RNeasy@Plant Mini Kit. The PrimeScript II 1st Strand cDNA Synthesis Kit (TaKaRa, Japan) and SYBR Premix Ex Taq II (TaKaRa, Japan) were used for reverse transcription reaction and qRT-PCR assay. Specific primers of the chosen genes were designed using Primer Premier 5.0 software (Additional file 2: Table S2). PCR products were verified by dissociation curves, and data were normalized with endogenous reference *tubulin* gene to obtain ΔCt values. Water was used as a negative and quality control, and each sample was measured in triplicate.

### Expression analysis of the flower-related genes in tissues and organs

The expression analysis of the flower-related genes in different tissues and organs was performed with qRT-PCR. Total RNA from the top and lateral buds (0.5–1 cm in length), the inner immature flowers (obtained from top bud when it grew to 1.5–3 cm in length), the corms, leaves, petals, stigmas, stamens and the remaining protective sheath of the full-bloom flowers, were extracted using an RNeasy@Plant Mini Kit and the following reverse transcription reaction and qRT-PCR assays were conducted according to the above description. The expression levels of flower-related genes in each sample were normalized to the *tubulin* gene to obtain ΔCt values. The top bud was used as a control sample, and the relative expression levels of target genes in the other samples were analysed using the 2^-ΔΔCt^ method: ΔΔCt = ΔCt other sample (Ct target gene- Ct *tublin*)- ΔCt control sample (Ct target gene- Ct *tubulin*).

### Time course expression analysis of flower-related genes during the flower development

Total RNA from four different stages of top buds from 20 g corms, including resting bud (1–2 mm in length), early stage of shoot growth (2–5 mm in length), late stage of shoot growth (5–10 mm in length), and stage of visually distinguishable flower organ formation (10–15 mm), were extracted using an RNeasy@Plant Mini Kit and the following reverse transcription reaction and qRT-PCR assays were conducted according to the above description.

### Data availability

The raw data were uploaded to Sequence Read Archive (SRA) (http://www.ncbi.nlm.-nih.gov/) with a reference of PRJNA528829.

## Results

### Long-length Transcriptome of saffron Crocus from PacBio Iso-seq

High-quality RNAs from top buds, tubers, pistils, stamens, petals and leaves of flowering saffron crocus were combined to acquire the PacBio Iso-seq libraries. Meanwhile, PacBio Iso-seq libraries of non-flowering saffron crocus were constructed using leaves, lateral buds, tubers, and top buds of non-flowering corms (20 g and 6 g). Multiple size-fractionated cDNA and cells (3 cells for 1–2 kb, 3 cells for 2–3 kb, 2 cells for > 3 kb) were prepared to construct flowering/non-flowering Iso-seq libraries separately. This approch avoids loading bias and obtaining more RNA sequences representing the gene expression profiles in flowering and non-flowering saffron crocus.

A total of 22.85 Gb of clean data were obtained from all sixteen cells with 1,325,207 raw polymerase reads and 23.9 billion nucleotides. After the adaptor and low-quality sequences were filtered, a total of 12,433,006 subreads were obtained, among which 7,178,336 and 5,254,670 subreads were in the libraries of flowering and non-flowering saffron crocus, respectively (Additional file 2: Table S3). High quality ROIs were further generated from CCS after filtering with full passes and accuracy. The numbers of ROIs from the flowering saffron crocus libraries were 224,710 for 1–2 kb, 199,782 for 2–3 kb, and 106,171 for 3–6 kb, respectively, which were more than those of the corresponding non-flowering saffron crocus libraries (179,712 for 1–2 kb, 73,160 for 2–3 kb, 52,904 for 3–6 kb) (Additional file 2: Table S4). In total, 394,653 (74.4%) and 252,850 (82.7%) full-length non-chimaera reads (FL non-chimaera, full-length reads with 3′ primer, 5′ primer and polyA reads after chimaera was filtered) were produced from ROIs of flowering and non-flowering saffron crocus libraries, respectively, with average lengths of 1223 bp, 2333 bp and 3512 bp in corresponding flowering saffron crocus libraries and 1188 bp, 2236 bp and 3322 bp in that of non-flowering saffron crocus libraries (Fig. 1b, Additional file 2: Table S4)).

After classification and correction by Clustering for Error Correction (CEC) and Quiver programs, 79,841 high-quality (Accuracy > 0.99) and 219,720 low-quality polished CCS were generated from ROIs. CCS were further corrected using the de novo assembly reads derived from Illumina RNA-seq. Ultimately, a total of 216,419 isoform level transcripts and 75,351 unigene transcripts were obtained after two-step CD-HIT classification of both flowering and non-flowering PacBio libraries. The length distribution of polished CCS, isoform and unigene is shown in Fig. 1c, with a majority of sequences ranging from 1 kb to 4 kb. The libraries of flowering and non-flowering saffron crocus were constructed separately, and the specific isoforms in each library and the differential expression profiles between flowering and non-flowering saffron crocus plants were obtained. The number of isoforms that expressed in both flowering and non-flowering saffron crocus was 174,369, while the number of isoforms that only expressed in flowering saffron crocus (30,188) were considerably more than those in non-flowering saffron crocus (11,862). These isoforms may provide a novel avenue to clarify the underlying molecular mechanism of floral development of saffron crocus.

Total 125 mRNAs derived from saffron crocus were reported on NCBI database at present. All the 75,351 full-length unigene transcripts were homologously aligned with them using BLAST. The results showed total 108 previously reported mRNAs were identified and matched with their highly homologous sequences in our data, with 86.4% coverage rate (Additional file 2: Table S5). Among them, 44 unigenes have a sequence identity of 99% or more and the identity of 88 unigenes were more than 95%, which suggested a full-length unigene database of saffron crocus with satisfactory coverage and accuracy was obtained in this study.

### Saffron Transcriptome of short-reads from Illumina RNA-seq

Fifteen Illumina RNA-seq libraries constructed from saffron crocus with different numbers of flowers (0–3) were sequenced to correct the polished CCS of PacBio Iso-seq and to quantify full length transcripts obtained from PacBio Iso-seq. After trimming process and screening with a high quality score, a total of 745 million clean reads were produced from all samples. Over 575 million short reads were successfully mapped back to the full-length of PacBio Iso-seq with an average mapping ratio of 77.2% (Additional file 2: Table S6), which suggested that the full-length transcripts derived from PacBio Iso-seq data method represented the majority of the genetic information of both flowering and non-flowering saffron crocus.

### Functional annotations

Databases such as NR, Swiss-Prot, KEGG (Additional file 3: Figure S2a), COG (Additional file 3: Figure S2b), and GO (Additional file 3: Figure S2c) were used to perform functional annotations to the 75,351 unigenes.

A total of 14,159 (21.9% of annotated unigenes) unigenes were associated with 34 pathways in KEGG pathway analysis. A high percentage of unigenes were assigned to “Translation” (10.3%) and “folding, sorting and degradation” (9.3%) of the genetic information process as well as “signal transduction” of the environmental information process (9.7%) (Additional file 3: Figure S2a).

A total of 64,562 unigenes (85.7%) were successfully matched to known sequences in at least one database. There were 99.5% matched unigenes in the NR database, 82.0% in SwissProt, and 72.0% in COG (Additional file 3: Figure S2d).

A total of 1193 GO terms were assigned to 33,117 unigenes (51.3% of annotated unigenes) with 454 biological processes, 159 cellular components and 580 molecular functions. In the class of biological processes, the top three GO terms were “metabolic process”, “cellular process”, and “single-organism process”. In the cellular component, “cell” was dominant and then “cell part” and “organelle”. In the class of molecular functions, a high percentage of the unigenes were enriched in “binding”, “catalytic activity” and “molecular function regulator” (Additional file 3: Figure S2c).

### CDS, SSR, and LncRNA prediction

**Fig. 2:**
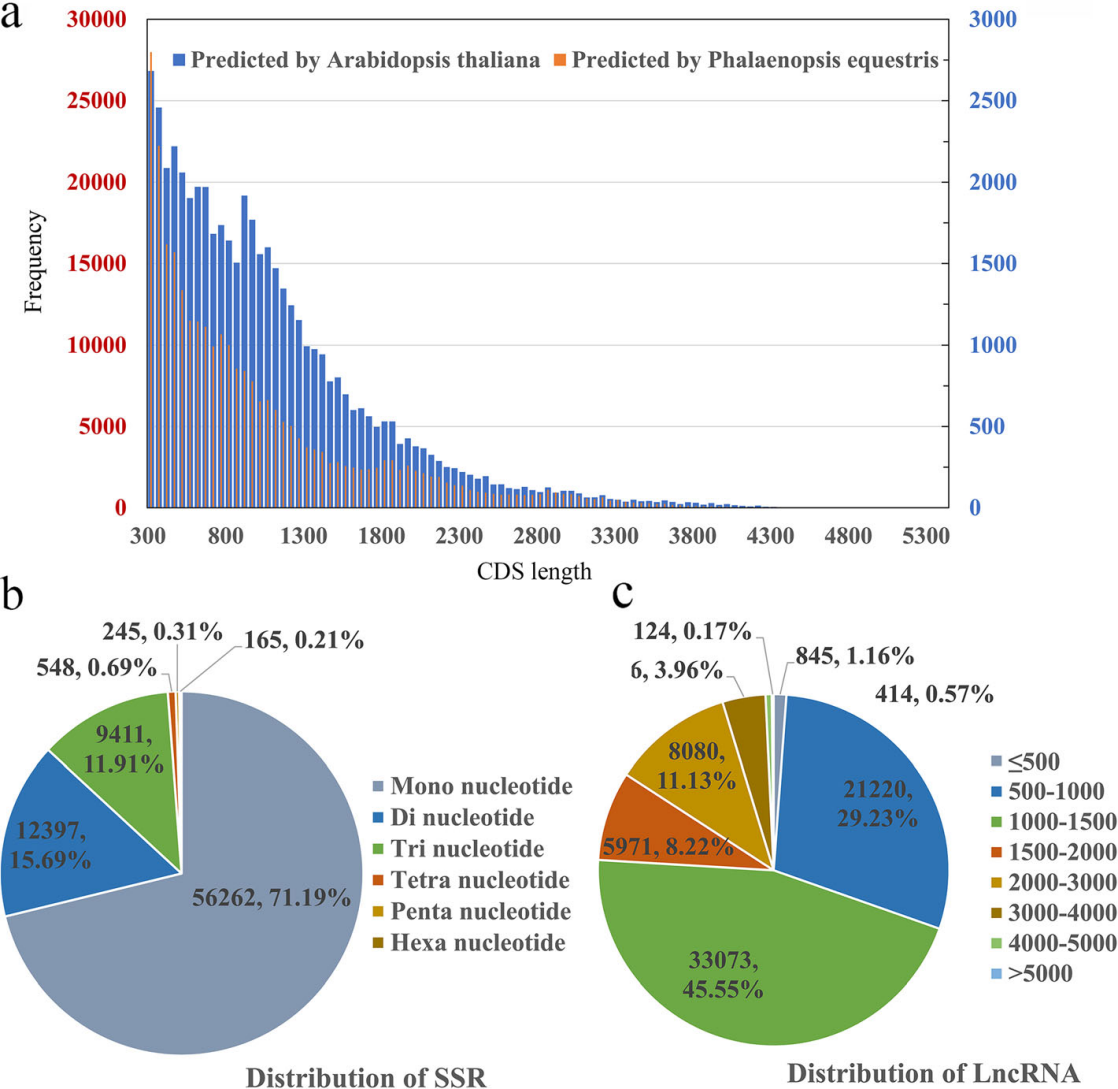
CDS, SSR and lncRNA analyses. **a**: Length distribution of CDS. **b**: Type distribution of SSRs. **c**: Length distribution of lncRNAs

The candidate coding sequence (CDS) in the PacBio transcript isoforms was analysed by retaining only open reading frames (ORFs ≥100 aa) using the ANGEL software. Both *Arabidopsis thaliana* and *Phalaenopsis equestris* genomes were used as the training sets. As shown in Fig. 2a, 50,197 CDS were obtained from the *Arabidopsis thaliana* genome with lengths ranging from 300 bp to 5400 bp and an average length of 1005 bp, while training with *Phalaenopsis equestris* genomes, ANGEL obtained a total of 289,377 predicted CDS with lengths ranging from 300 bp to 5400 bp and an average length of 1081 bp. Because saffron crocus is more closely related to orchids, more comprehensive information on encoded proteins would be obtained using orchid as the training set.

SSRs, also known as microsatellite DNAs, have a tandem repeat motif of 1–6 bp in length. The most common motifs are dinucleotide repeats, such as (CA) n and (TG) n. The characters of high polymorphism (mainly due to the difference in the number of tandem motifs), stability, and reliability enable it to be an ideal molecular marker that is widely used in such applications as genetic map construction, quantitative trait locus (QTL) mapping and genetic diversity assessment. A total of 79,028 SSRs were identified in 34,895 unigenes (46.3% of total unigenes), including six types of SSR: mono-nucleotide (56,262, 71.2% of all SSRs), di-nucleotide (12,397, 15.7%), tri-nucleotide (9411, 11.9%), tetra-nucleotide (548, 0.7%), penta-nucleotide (165, 0.2%), and hexa-nucleotide (245, 0.3%) (Fig. 2b); among them, 28,993 SSRs present in compound formation.

The PLEK workflow of lncRNA-pipeline was used to discriminate between coding and non-coding transcripts and then identify lncRNAs using PacBio data from species with no reference genome. To obtain more putative lncRNA candidates for saffron crocus, 216,419 isoform transcripts were used to predict lncRNAs in this study. A total of 72,603 (33.5%) PacBio non-coding transcripts were obtained and the length ranged from 194 bp to 6860 bp with an average length of 1367 bp. Similar to other species, the length abundance is concentrated at 500–1500 bp (54,296, 74.8%) (Fig. 2c)**.**

### Alternative splicing analysis and validation

**Fig. 3:**
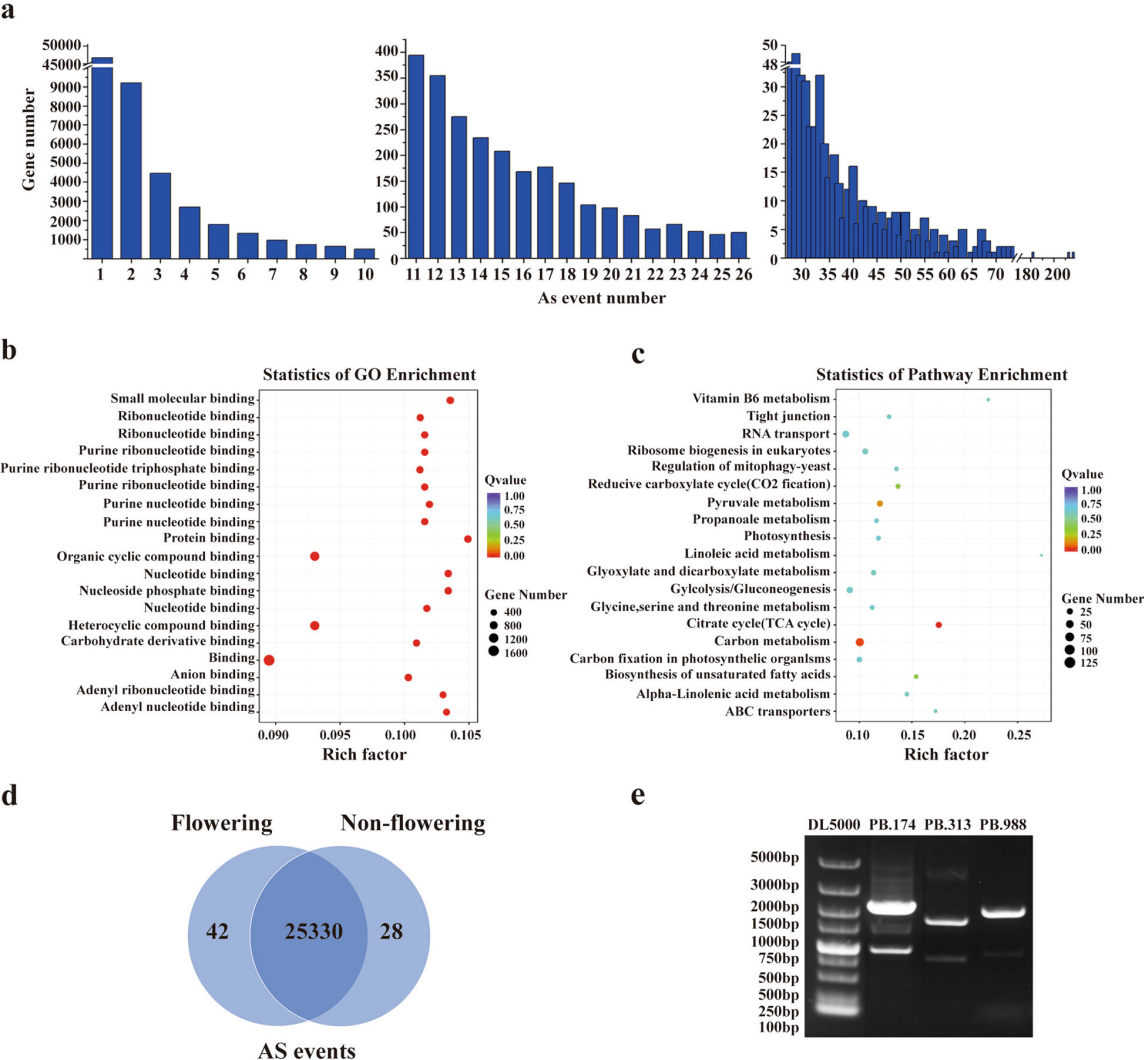
Alternative splicing (AS) analysis and validation. **a**: The distribution of AS event numbers. **b**: GO enrichment analysis of AS genes. **c**: KEGG enrichment analysis of AS genes. **d**. Venn diagram of AS events presented in PacBio-Seq libraries of the flowering and non-flowering saffron crocus. **e**. The gel electropherogram showed AS events of PB.174, PB.313, and PB.988

Most mRNA precursors of eukaryotic genes produce only one mature mRNA that is thus translated to only one molecular protein. However, some mRNA precursors can produce different mRNA splice isoforms by different splicing sites, which is known as alternative splicing (AS). AS is an important mechanism of regulating gene expression and producing proteome diversity. At present, it is still challenging to reconstruct full-length splice isoforms using Illumina-based transcriptome assembly [37, 38]. Splice isoforms with multiple introns make it difficult to identify alternative splicing using short read lengths, which were constrained by cufflink-based assemblies. One of the most important features of PacBio Sequencing is the ability to identify alternative splicing by directly comparing isoforms of the same gene without de novo assembly and thus avoiding artificial mistakes. Among the 75,351 unigenes identified in saffron crocus, 33.7% (25,400) have two or more isoforms. The number of AS events ranged from 2 to 217, and the distribution of AS events is shown in Fig. 3a. GO enrichment analysis showed that these AS genes were enriched in 120 pathways, with the top three being “Binding”, “Heterocyclic compound binding” and “Organic cyclic compound binding” (Fig. 3b). It was interesting that the top 20 pathways were involved in various binding activities of molecular function ontology and a few AS genes were annotated with functions of catalytic activity and transport activity (Additional file 2: Table S7). Only two KEGG pathways, “Citrate cycle (TCA cycle)” and “Carbon metabolism”, were significantly enriched by AS genes (Fig. 3c).

Furthermore, different splicing events between flowering and non-flowering saffron crocus were screened in PacBio-Seq libraries because AS is highly tissue-specific. A total of 42 AS events were identified in the PacBio-Seq libraries of flowering saffron crocus only, while 28 AS events were found in those libraries of non-flowering one (Fig. 3d, Additional file 2: Table S8). The AS events of three randomly selected unigenes, PB.174, PB.313 and PB.988, were validated using RT-PCR. Four, three, and two AS events were shown in the gel electropherogram (Fig. 3e), and the correctness of the amplification was proved by sequencing of the PCR products.

### Differentially expressed Unigenes between flowering and non-flowering saffron Crocus corms

The expression levels of all unigenes from PacBio-Seq libraries in five groups of saffron crocus corms with different flowering phenotypes were quantified using an FPKM method based on the results of Illumina RNA-seq. In general, when FPKM < 0.1, the gene does not express; 0.1 ≤ FPKM < 3.75, the gene is considered to be expressed at a low level; 3.75 ≤ FPKM < 15, the gene is at a median level; FPKM ≥15, the gene is expressed at a high level. RNA-Seq2 software was used to screen differentially expressed unigenes between flowering and non-flowering saffron crocus corms, and the threshold of significance was determined as *p*-value < 0.05, false discovery rate (FDR) < 0.01 and log2 fold-change < − 1 or > 1.

**Fig. 4:**
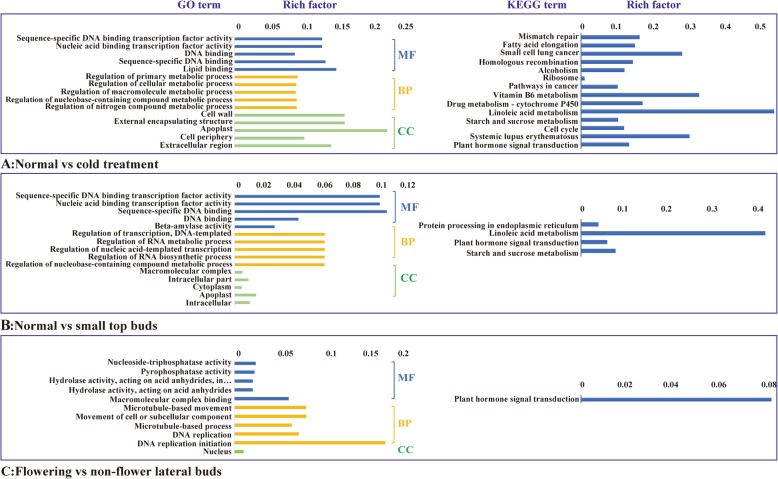
GO and KEGG enrichment analyses of DEGs between flowering and non-flowering saffron crocus. **a**: GO and KEGG enrichment analyses of DEGs between flowering top buds and matched non-flowering cold-treated top buds. **b**: GO and KEGG enrichment analyses of DEGs between flowering top buds and non-flowering top buds of small corms. **c**: GO and KEGG enrichment analyses of DEGs between flowering lateral buds and matched non-flowering lateral buds

The three top bud samples of flowering saffron crocus were compared to the three matched samples of non-flowering saffron crocus with cold treatment. A total 1677 DEGs (Additional file 2: Table S9), 70.8% (1187) of which were up-regulated, were screened with log_2_ fold-changes ranging from − 9.4 to 12.6. All the DEGs were significantly enriched in 109 GO terms (q-value < 0.05), in which the function of the top five pathways was on binding and transcription factor activity with nucleic acid. These proteins play critical roles as transcription factors or at the transcriptional level in low temperature stress and flowering signalling pathways (Fig. 4a).. In addition, 11 unigenes were specifically highly expressed in three top buds of non-flowering saffron crocus with cold treatment while no expression was observed in any of the matched flowering samples. In contrast, 238 unigenes were highly expressed in three flowering samples exclusively (Additional file 2: Table S10). The DEGs with high fold-changes and strict consistency in biological repeat samples were ideal candidates for further larger-scale validation.

A total of 1086 significant DEGs (Additional file 2: Table S9), 81.7% (887) of which were up-regulated, were identified in top buds between flowering saffron crocus and non-flowering saffron crocus of 6 g corms, with log_2_ fold-changes ranging from − 14.2 to 10.6. A total of 141 GO terms were clustered, and the top 5 pathways in each main ontology are shown in Fig. 4b. Within the cellular component ontology, the DEG-coding proteins were mainly located in the “macromolecular complex”, “intracellular part” and “cytoplasm”. For the biological process ontology, DEG-coding proteins were mainly involved in the transcription processes, such as “regulation of transcription, DNA-templated”, “regulation of RNA metabolic process” and “regulation of nucleic acid-templated transcription”. In terms of molecular function ontology, DEG-coding proteins were binded with nucleic acid and functioned as transcriptional regulation factors (Fig. 4b). In addition, 70 and 32 unigenes were exclusively highly expressed in all samples of large corms of flowering saffron crocus and small corms of non-flowering saffron crocus, respectively (Additional file 2: Table S11).

hree paired samples of lateral buds from the same corms of flowering and non-flowering saffron crocus were collected to screen key genes that regulate the flowering of lateral buds. A total of 267 DEGs (Additional file 2: Table S11), 83.5% (223) of which were down-regulated, were identified with log_2_ fold-changes ranging from − 8.5 to 9.4. All 267 DEGs were assigned to 75 GO terms. The only GO terms in the cell component ontology was “nucleus”. The most highly enriched pathway in biological progress ontology was “microtubule- based movement” (GO level 5), which was in the sub-grade GO level of both top 2 (“movement of cell or subcellular component”, GO level 4) and top3 (“microtubule-based process”, GO level 4) pathways, followed by the pathway of “DNA replication”. In the molecular function ontology, most unigenes were highly enriched in a series of pathways from GO level 4 (hydrolase activity, acting on acid anhydrides) to GO level 7 (Nucleoside-triphosphatase (NTPase) activity). NTPases activation may regulate the nuclear export of mRNA and are known to be involved in defense signaling pathways and apoptosis regulation [39] (Fig. 4c)**.**. In addition, 15 unigenes (Additional file 2: Table S11) were highly expressed in three lateral buds of non-flowering saffron crocus specifically but were not expressed in lateral buds of flowering saffron crocus. Only 7 unigenes were exclusively highly expressed in lateral buds of flowering saffron crocus (Additional file 2: Table S12). These 22 unigenes could be preferentially used to explore the molecular mechanism of the flowering process of lateral buds.

As for KEGG pathway analysis, although the DEGs involved in each experiment are different, “plant hormone signal transduction” was assigned by all the three comparable experiments, which even is the only pathway enriched in flowering vs. non-flower lateral buds (Fig. 4c). The changes of flowering phenotype caused by nutritional status (normal vs. small top buds), low temperature stress (normal vs. cold treatment) or natural variation (flowering vs. non-flower lateral buds) may be involved in different pathways, but they shared the same regulation network of plant hormones, including the response to hormone auxin, abscisic acid and cytokinin (for example, auxin-responsive protein IAA10-like, probable indole-3-acetic acid-amido synthetase GH3.5, auxin transporter-like protein 3, abscisic acid receptor PYL8-like, two-component response regulator ARR8).

Both DEGs of normal vs. small top buds and normal vs. cold treatment were assigned to the KEGG pathways of “starch and sucrose metabolism” and “linoleic acid metabolism”. It has been recognized for long time that temperature could modify carbohydrate metabolism of some plants by regulating starch and sugar levels [40]. Furthermore, altered carbohydrate metabolism impacted plant biomass production, as well as flower development. Actually, altering the expression of multiple sucrose metabolism-related genes in tobacco, such as UGPase, SuSy and SPS which were also found in DEGs of saffron crocus, not only enhanced primary growth but also altered flower morphology [41]. Based on the fact that flower numbers of saffron crocus were heavily depended on temperature and corm weight, the results of KEGG enrichment suggested that carbohydrate metabolism may play important roles in flower development of saffron crocus.

**Fig. 5:**
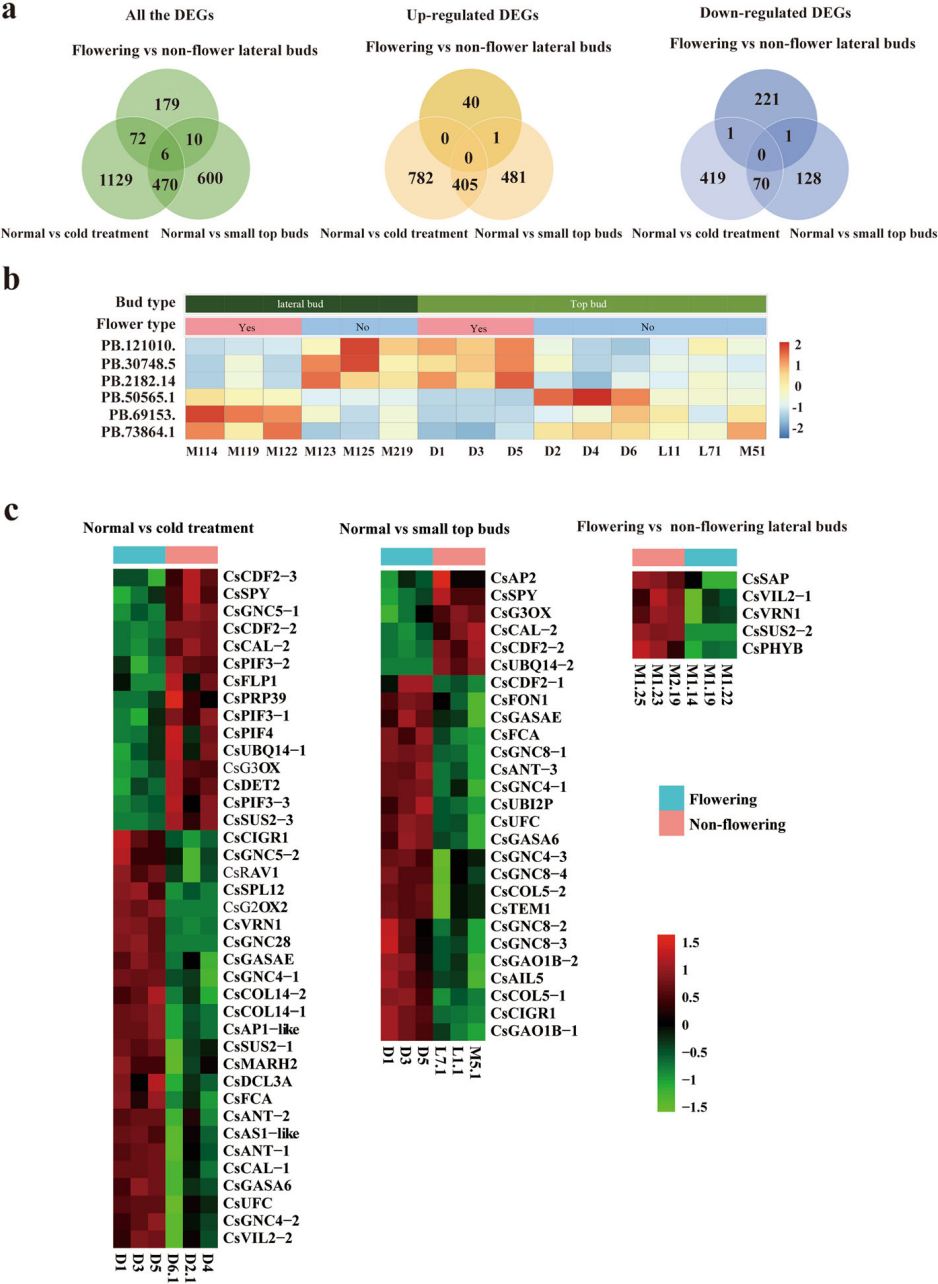
Expression pattern analyses of all the DEGs in three comparable experiments. **a**: Venn diagram of distributions of all the DEGs (left), upregulated DEGs (middle) and downregulated DEGs (right) in the three comparable experiments. **b**: The heatmap of the expression patterns of six unigenes shared in three comparable experiments (the group of flowering lateral buds consisted of M114,M119 and M122; the group of non-flowering lateral buds consisted of M123,M125 and M219; the normal group consisted of D1,D3 and D5; the group of cold treatment consisted of D2,D4 and D6; the group of small top buds consisted of L11, L71 and M51). **c**: Heatmap of the expression patterns of 62 flower-related DEGs in different comparable groups

There are many endogenous or exogenous factors affecting the flowering process of saffron crocus and driving different gene expressions. Genes shared in different pathways were initially speculated to regulate the flowering process of saffron crocus. All up and down regulated DEGs from the three comparable experiments (normal vs. cold-treated corm; big vs. small corm; top vs. lateral bud) were analysed using Venn software. Among all the DEGs, only six unigenes were shared in three groups. Unexpectedly, neither shared gene was identified in the upregulated nor downregulated DEGs (Fig. 5a). All the upregulated DEGs in the lateral buds of flowering saffron crocus were downregulated in the top buds of flowering saffron crocus and vice versa (Fig. 5b). Similar results were found in the DEGs shared in the two groups. For example, 78 shared DEGs (72 in two groups and 6 in three groups) were found in the lateral bud group and cold-treated top bud group, while only one shared DEG had consistent expression in both groups (Fig. 5a). Almost all shared DEGs had consistent expression in cold-treated top buds and normal/small top buds (475/476). The results suggested that there were different networks in regulating the flowering process of lateral buds of saffron crocus, and these networks may have been shared in the top buds.

### Identification and expression analysis of flower-related DEGs in saffron Crocus

A total of 62 DEGs were functionally annotated as putative flower-related genes (Additional file 2: Table S13) and we analysed the expression patterns of all the transcripts using RNA-seq data (Fig. 5c). We identified 39 flower-related genes (assigned with predicted gene names) in the normal vs cold treatment group, 24 of which were significantly highly expressed in flowering corms. CsVRN (*B3 domain-containing transcription factor*) is a key gene involved in the vernalization pathway, which may affect flower development when the saffron crocus was treated with cold.

In the normal vs. small top buds group, 33 flower-related genes were identified and interesting, *putative flowering time control protein* (CsFCA) and *squamosa promoter-binding-like protein 12* (CsSPL12), which are involved in autonomous and age pathways, were significantly highly expressed in the larger corms. The decreasing expression levels of CsFCA and CsSPLs may partly explain why the small corms (weight less than 6 g) cannot bloom. Furthermore, SPL12, strongly regulated together with CONSTANS (CO), were negatively regulated by miR156b and miR156h, suggesting microRNAs biogenesis or microRNA alteration maybe involved in the process of floral initiation [42].

We identified fourteen shared flower-related DEGs in normal vs cold treatment and normal vs. small top bud groups. Most of them (6/14) were involved in the gibberellin pathway, including putative *gibberellin 3-beta-dioxygenase 1-like* (CsG3OX), *gibberellin-regulated protein 6-lik*e (CsGASA6) and *gibberellin-regulated protein 14* (CsGASAE) .

Only five flower-related DEGs were identified in the flowering vs. non-flowering lateral bud groups and all of them were significantly lower in the flowering samples. There may be some novel genes, and even new regulatory pathways trigger the lateral bud bloom.

**Fig. 6:**
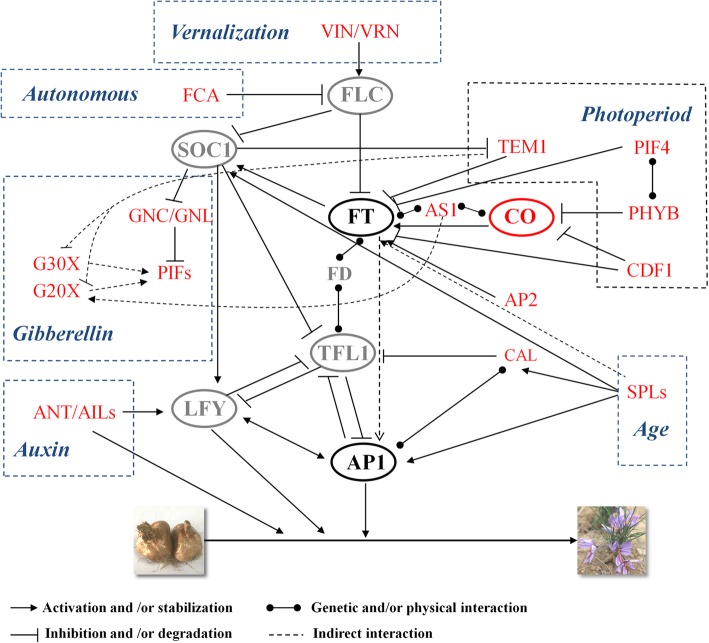
Schematic diagram of the flowering gene regulatory network in saffron crocus. Genes marked in red are identified first in this study. Genes marked in grey and inside circles are important key flower-related genes that have not been identified at present

Because few flower-related gene sequences were submitted to the public database, among the 62 flower-related DEGs that we identified in this study, only PB.59337.3 (putative *Apetala1-like MADS-box transcription factor*, assigned as CsAP1-like) was matched to *Crocus sativus*. To clarify the flower-related gene regulatory network of saffron crocus, we plotted the schematic representation based on the known flowering gene network of *Arabidopsis thaliana* (Fig. 6): the flowering process of saffron crocus, similar to most other species, was involved in vernalization, gibberellins, photoperiod, autonomous and age pathways.

### Validation of novel flower-related genes in larger-scale samples using real-time RT-PCR

**Fig. 7:**
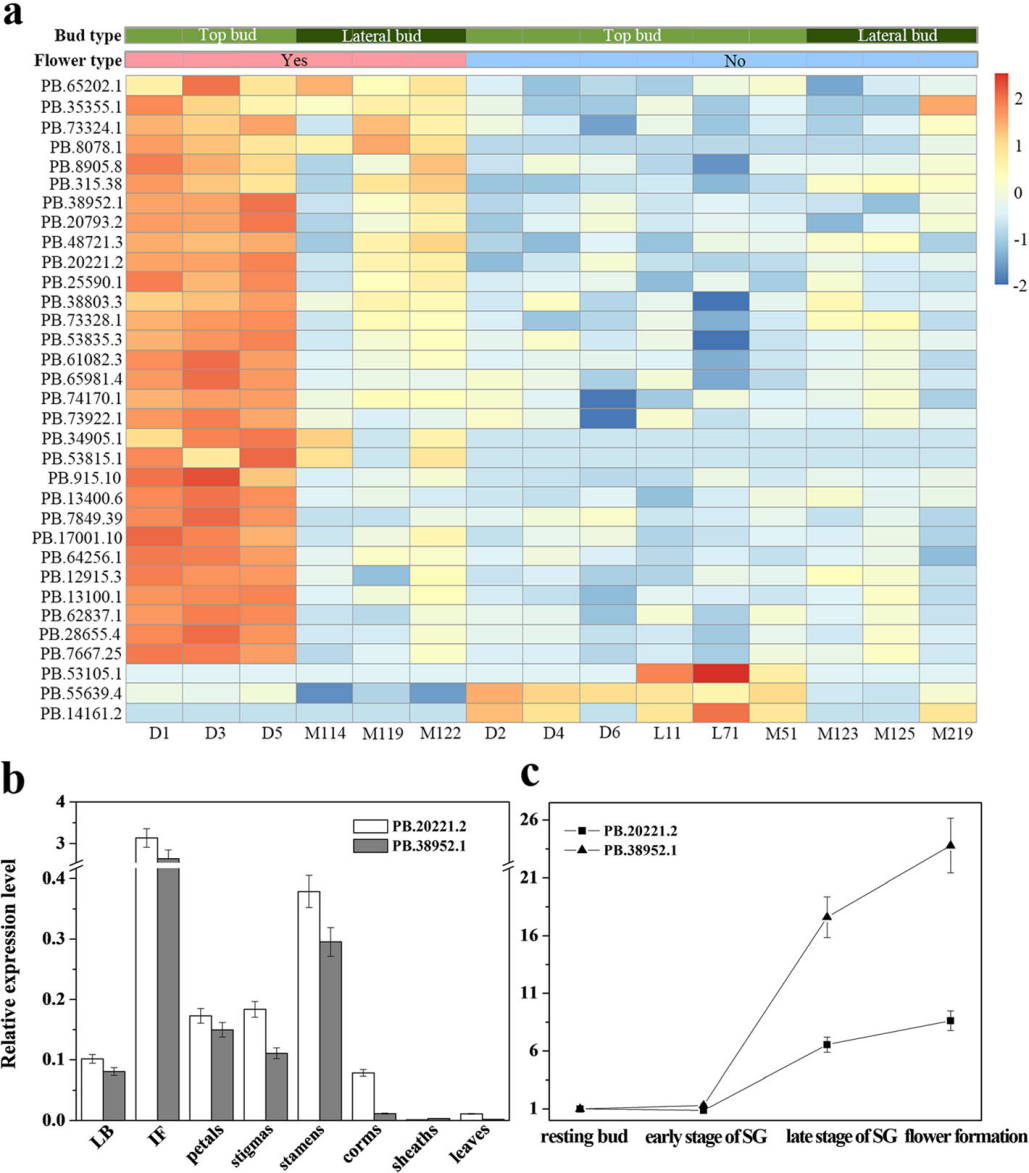
Validation of novel flower-related genes using RT-PCR. **a**: heatmap of the expression patterns of 33 DEGs between flowering samples and non-flowering samples. **b**: The relative expression levels of PB.20221.2 and PB.38952.1 compared to the top buds were performed on lateral buds (LB), immature flowers (IF), petals, stigmas, stamens corms, protective sheath and leaves. **c**: The relative expression levels of PB.20221.2 and PB.38952.1 compared to the resting top buds were performed on the top buds during the period of flower development

Except for the 62 genes that are homologous to known flowering gene sequences in public databases, we attempted to select and validate more novel flower-related genes in DEGs. Therefore, the fifteen samples were reclassified into six flowering samples (three flowering top buds and three flowering lateral buds) and nine non-flowering samples (three cold-treated top buds, three small top buds and three lateral buds) to identify DEGs using RNA-seq2 software with the same significance threshold as above. Thirty-three DEGs were screened out, and only 3 unigenes were downregulated in the flowering group (Fig. 7a). Though the differences in DEG expression levels in top buds were more significant than those in lateral buds, the DEGs related to flowering processes were likely involved in downstream pathways or common nodes in flowering regulatory networks.

Ten DEGs related to the flowering process were selected to validate the key flowering genes using an additional 30 saffron crocus buds. The biological parameters of saffron crocus buds were collected, including the type of bud (top or lateral) number of flowers (0–2), length of bud (2 mm - 5 cm), and fresh corm weight. Real-time RT-PCR was used to assay the expression levels of 10 unigenes in the 30 buds of saffron crocus and the correlation between relative expression levels of each gene (ΔCT) and biological parameters of saffron crocus buds were analysed using a Pearson (length of bud, fresh corm weight) or a Spearman method (type of bud, number of flower).

The expression pattern is shown in Additional file 4: Figure S3 and the results of the correlation analysis are shown in Table 1. Both PB.20221.2 and PB.38952.1 were significantly correlated with flower status and showed high expression in flowering plants (*p* = 0.004, *r* = 0.52; *p* = 0.023, *r* = 0.41). Global alignment analysis (https://www.ncbi.nlm.nih.gov/igblast/) showed that PB.20221.2 and PB.38952.1 share 68% homology, and BLASTx showed that both genes were predicted to be heat stress transcription factor A-2b-like genes. In addition, some unigenes may contribute to the physiological development of saffron crocus; for instance, PB.53815.1 expression was correlated with the length of the buds (*p* = 0.04, *r* = 0.39), while PB.315.38 expression was correlated with the fresh weight of the corm (*p* = 0.0 01, *r* = 0.50).

**Table 1:** Correlation analyses between expression levels of flower-related candidate genes and biological parameters of saffron crocus buds

Gene ID	Bud type		Flower type		Bud length		Corm weight	
	*p*-value	r	*p*-value	r	*p*-value	r	*p*-value	r
PB.20221.2	0.92	0.02	0.004*	−0.52	0.20	0.24	0.67	−0.08
PB.38952.1	0.62	−0.09	0.023*	−0.41	0.26	0.21	0.08	−0.33
PB.73324.1	0.647	0.09	0.187	−0.25	0.14	0.28	0.61	0.10
PB.53835.3	0.20	−0.24	0.563	−0.11	0.64	0.09	0.38	−0.17
PB.35355.1	0.46	0.14	0.97	−0.01	0.80	−0.05	0.90	−0.02
PB.64256.1	0.39	−0.16	0.74	−0.06	0.89	0.03	0.37	−0.17
PB.65202.1	1.00	0.00	0.23	−0.23	0.20	−0.24	0.79	−0.05
PB.38803.3	0.53	−0.12	0.61	−0.10	0.35	0.18	0.72	−0.07
PB.53815.1	0.72	0.07	0.46	−0.14	0.04**	−0.39	0.10	−0.31
PB.315.38	0.41	−0.16	0.26	−0.21	0.66	−0.08	0.001**	−0.563

*, ** denote the significant correlation at the 0.05 level analyzed by Spearman method and Pearson method, respectively

### Expression analysis of the novel flower-related genes in tissues and organs

The analysis of PB.20221.2 and PB.38952.1 was performed on the buds, corms, immature flowers, leaves, petals, stigmas, stamens and the remaining protective sheath (Fig. 7b). Compared to top buds, both PB.20221.2 and PB.38952.1 were highly expressed in the immature flowers (mean fold change = 3.13 and 2.64, respectively). PB.20221.2 and PB.38952.1 were relatively less expressed in lateral buds (mean fold change = 0.10 and 0.08, separately), petals (mean fold change = 0.17 and 0.15, separately), stigmas (mean fold change = 0.18 and 0.11, separately) and stamens (mean fold change = 0.38 and 0.29, separately). PB.38952.1 was weakly expressed in corms (mean fold change = 0.01), leaves (mean fold change = 0.002) and protective sheath (mean fold change = 0.003), while PB.20221.2 could be detected in leaves (mean fold change = 0.01) and corms (mean fold change = 0.08) with notable low expression levels but not in protective sheath (Ct > 35).

Both genes were relatively highly expressed in immature flowers, which was similar to the expression pattern of previously isolated flower-related genes in saffron-CsatFT2, CsatFT3, CsatCEN/TFL1 [10, 13] and AP1/FUL In addition, PB.20221.2 and PB.38952.1 were relatively more highly expressed in the various tissues of flower organs (petals, stigmas, stamens) than in other organs (corms, leaves, sheath), which suggested a potential function in flower development.

### Time course expression analysis of novel flower-related genes during flower development

To understand the variations of PB.20221.2 and PB.38952.1 expression at different stages of floral organ development, we performed a quantitation of the transcripts in top buds from the dormant period to the start of the flowering season (Additional file 5: Figure S4). As shown in Fig. 7c, PB.20221.2 showed a fluctuation in the expression levels with the maximum expression during the stage of visually distinguishable flower organ formation (mean fold change = 8.62). The transcripts of PB.38952.1 remained constant from the stage of resting bud to the early stage of shoot growth (mean fold change = 1.31), began to accumulate at the late stage of shoot growth (mean fold change = 17.60) and largely increased at the stage of visually distinguishable flower organ formation (mean fold change = 23.79).

Taken together, the expression data in different tissues, organs and different floral development stages suggest that PB.20221.2 and PB.38952.1, probably had the function of promoting floral organ formation and development and were new flower-related genes worthy of further study in the regulatory network of the flowering pathway. The nucleotide sequences of PB20221.2 and PB38952.1 were carried out alignment with Web BLAST-blastX on NCBI database to characterize them and the listing protein sequences were downloaded and screened for Phylogenetic analysis. The deduced amino acid sequences of PB20221.2 and PB38952.1 were aligned with the selected listing protein sequences using the multiple sequence alignment program ClustalX and phylogenetic tree were constructed with MEGA-X software using neighborjoining-method with 1000 bootstrap replicates. Aligning with homologous sequences from 38 different species, Phylogenetic studies revealed that PB20221.2 and PB38952.1 belonged to heat stress transcription factor family (HSF) and clustered in HSF-A2b sub-family (Additional file 6: Figure S5), indicating they were probably associated with the regulation of heat stress in flowering process of saffron crocus and further studies were needed to investigate their specific functions.

## Discussion

Comprehensive, high-quality, full-length transcriptome sequences were presented for saffron crocus using SMRT technology in this study. A total of 75,351 full-length saffron crocus unigenes were identified with an average length of 2049 bp in the flowering saffron crocus library and 1802 bp in the non-flowering library. Among these unigenes, 64,562 (85.7%) were functionally annotated. Based on RNA-seq data, Baba et al. identified 64,438 transcripts with an average length of 609 bp and a functional annotation ratio of 58.5% (37,696) [19]. Subsequently, Jain et al. found 105,269 transcripts with an average length of 1047 bp [20], and 54% of them were functionally annotated. Basically, illumina RNA-seq can obtain higher coverage of genome and more transcripts than SMRT-Seq due to relatively higher sequencing depth. Our results further confirmed that SMRT-seq was more effective in recovering full-length transcripts [22]. The higher functional annotation ratio of unigenes in our study suggested that SMRT-seq data could provide higher accuracy and more effective information on saffron crocus transcriptomes.

Based on the PacBio-seq platform, 79,028 SSRs were identified in 34,895 unigenes (46.3% of total unigenes) in this study, which was considerably more than that predicted in the Illumina platform (16,721 SSRs identified in 13,407 transcripts). As popular molecular markers, SSRs are widely used for determining genetic variations, and the most important advantage is locus specificity, which is highly suitable for allopolyploid species, such as saffron crocus [43]. It should be noted that the status of genetic variation in saffron crocus is still controversial since several studies provided contrasting results [44]. Thus, the novel candidate SSRs of saffron crocus may provide pools to explore new alternative SSRs. In addition, the comprehensive CDS, lncRNAs and AS prediction of saffron crocus were reported for the first time in this study.

PacBio Iso-Seq libraries of flowering and non-flowering saffron crocus were constructed separately to identify as many candidate genes as possible without missing flowering suppressor genes that are expressed in non-flowering corms only. More unigenes were identified exclusively in flowering saffron crocus (30,188) than in non-flowering saffron crocus (11,862). This result suggested that saffron crocus plants might have additional genes to regulate floral development. The result might also indicate that suppressor genes for floral development may exist in some tissues of non-flowering saffron crocus only. The individual PacBio Iso-Seq library of flowering and non-flowering saffron crocus facilitated the clarification of specific AS in each library. AS functioned to regulate gene expression by introducing a premature termination codon andled to special RNA splicing isoform degradation. Increased AS was found in various plants with the application of high-throughput sequencing while the biological significance of most AS events was not determined. Recent research has demonstrated the direct function of AS in controlling the initiation and timing of flowering [45]. A total of 25,400 AS events in the full-length transcriptome were identified, and 42 and 28 AS events were identified in the flowering and non-flowering libraries, respectively.

The underlying molecular mechanism for lateral buds of saffron crocus to flower was possibly different from top buds. The GO and KEGG pathway analyses of DEGs in lateral buds suggested potential genetic variation of flowering lateral buds, based on the enriched pathways of “cell cycle”, “meiosis”, “mismatch repair”, “DNA replication” and “nucleoside-triphosphatase activity”. The DEGs in top buds were found to function at transcriptional levels as well as in the signal pathways responding to hormones, nutrients, and other environments. Furthermore, the DEGs in lateral buds were completely different from top buds. The six shared DEGs were expressed in opposite trends between lateral and top buds. Saffron crocus multiplies by means of corms, but corm multiplication does not generate genome variation, except for occasional somatic mutations. However, phenotypic variations of flowers have been frequently observed in saffron crocus, as demonstrated by different numbers of stigmas, different aspects of tepals or blooming in lateral buds [30, 46]. As the genetic variability in saffron crocus is still under debate, whether the phenotype variations are influenced by genetic variability or the environment warrants further investigation [47].

In this study, PB.20221.2 and PB.38952.1 were significantly correlated with flowering quantities and were suggested as flower-related genes. Phylogenetic analysis showed both genes encoded for HSF-A2b. Heat stress transcription factor family are the terminal components of a signal transduction chain mediating the activation of genes responsive to heat stress, including more than twenty members and classified as A, B and C in plants. HSF-A2 is a strong transcription activator through interacting with heat shock proteins (HSPs) during long-term heat stress [48]. Heat stress-regulated flowering was recently recognized as a floral transition pathway [49], together with the fact that HSP family was required for floral meristem formation [50]. Thus, as the central regulator of HSP expression, HSF-A2 may play important roles in the induction of inflorescence meristem formation. In fact, all the corms of saffron crocus can blossom only after enough heat accumulation of high temperature higher than 23 °C, which also consistent with our results.

In conclusion, full-length transcriptomes of flowering and non-flowering saffron crocus were obtained using a combined NGS short-read and SMRT long-read sequencing approach. This method enables the generation of predicted comprehensive databases of AS, lncRNA, SSR, CDS, and DEGs in samples with phenotypic differences in flowering traits. These results were used to further identify genes related to flowering, including 62 genes homologous to known flowering gene sequences in public databases and novel flower-related genes,such as PB.20221.2 and PB.38952.1. Our study represents a first step to establish a reference full-length transcriptome for future studies of the gene atlas of saffron crocus and other species in the Iridaceae family. In the future, these genes, which are closely related to the number of flowers selected out in this study, maybe used as biomarkers for screening multi-flowering varieties and monitoring the optimal environmental conditions in field production.

## Supplementary information

**Additional file 1: Figure S1.** Morphology of the saffron samples; a: the whole saffron plant with flowers on both top and lateral buds. b: The sites of top buds and lateral buds of saffron crocus plants. c:Sampling sites of buds of saffron crocus. d: The flowering top bud (right) and paired cold-treated non-flowering top bud (left) of 20 g corms, which were spited into two parts and cultivated at room temperature or 10 °C separately. e: the pistils of the flower. f: the leaves of saffron crocus. g: the stamens of saffron crocus.

**Additional file 2: Table S1.** Primers of Unigenes for AS validation. **Table S2.** Primers of differentially expressed Unigenes for real-time qRT- PCR analysis. **Table S3.** PacBio Iso-seq data statistics. **Table S4.** Summary of reads of insert (ROIs). **Table S5.** Homology Analysis between mRNA sequence on NCBI and full-length unigene. **Table S6.** Sequencing data statistics of Illumina RNA-seq. **Table S7.** GO enrichment analysis of AS genes. **Table S8.** Specific AS events in flowering saffrons and non-flowering saffron crocus. **Table S9.** Number of DEGs in flowering and non-flowering saffron crocus. **Table S10.** DEGs only expressed in flowering top buds or non-flowering cold-treated top buds. **Table S11.** DEGs were only expressed in flowering top buds or non-flowering top buds of small corms. **Table S12.** DEGs were only expressed in flowering or non-flowering lateral buds. **Table S13.** Flower-related DEGs in three comparable experiments.

**Additional file 3: Figure S2.** Functional annotations of 75,351 unigenes. a: KEGG pathway analysis of all unigenes. b: COG pathway analysis of all unigenes. c: GO pathway analysis of all unigenes. d: Venn diagram of functional annotations of NR, Swiss-Prot, KEGG, COG and GO databases. (TIF 19440 kb)

**Additional file 4: Figure S3.** The heatmap shows the expression patterns of ten DEGs in 30 saffron crocus corms.

**Additional file 5: Figure S4.** The top buds of saffron corms during the time-course from the dormant period to the start of the flowering season. a: resting bud; b: early stage of shoot growth; c: late stage of shoot growth; d: stage of visually distinguishable flower organ formation.

**Additional file 6: Figure S5.** Phylogenetic analysis of a representative subset of 38 amino acid sequences belonging to the HSF family and the HSF-like deduced proteins isolated from saffron crocus (PB20221.2 and PB38952.1). Phylogenetic relationships of the sequences were examined using the neighbor-joining method.

## Data Availability

The raw data were uploaded to Sequence Read Archive (SRA) (http://www.ncbi.nlm.-nih.gov/) with a reference of PRJNA528829.

## Abbreviations

AS: Alternative Splicing
CCS: Circular-Consensus Sequence
CDS: Coding Sequences
COG: Clusters of Orthologous Groups
DRCLR: Duplication-Removed and Corrected Long Reads
FPKM: Fragments Per Kilobase of transcript per Million mapped reads
GO: Gene Ontology
KEGG: Kyoto Encyclopedia of Genes and Genomes
LncRNA: Long non-coding RNA
NGS: Next Generation Sequence
NR: NCBI non-Redundant
ORF: Open Reading Frames
ROIs: Reads of Inserts
SMRT: Single Molecule Real-Time
SRA: Sequence Read Archive
SSR: Simple Sequence Repeats
UCS: Unpolished Consensus Sequences
