# Supplementary figures and images for: Single-molecule real-time transcript sequencing identified flowering regulatory genes in Crocus sativus
Source: BMC Genomics. 2019 Nov 14;20:857. doi: 10.1186/s12864-019-6200-5 (PMC6854690; doi:10.1186/s12864-019-6200-5)

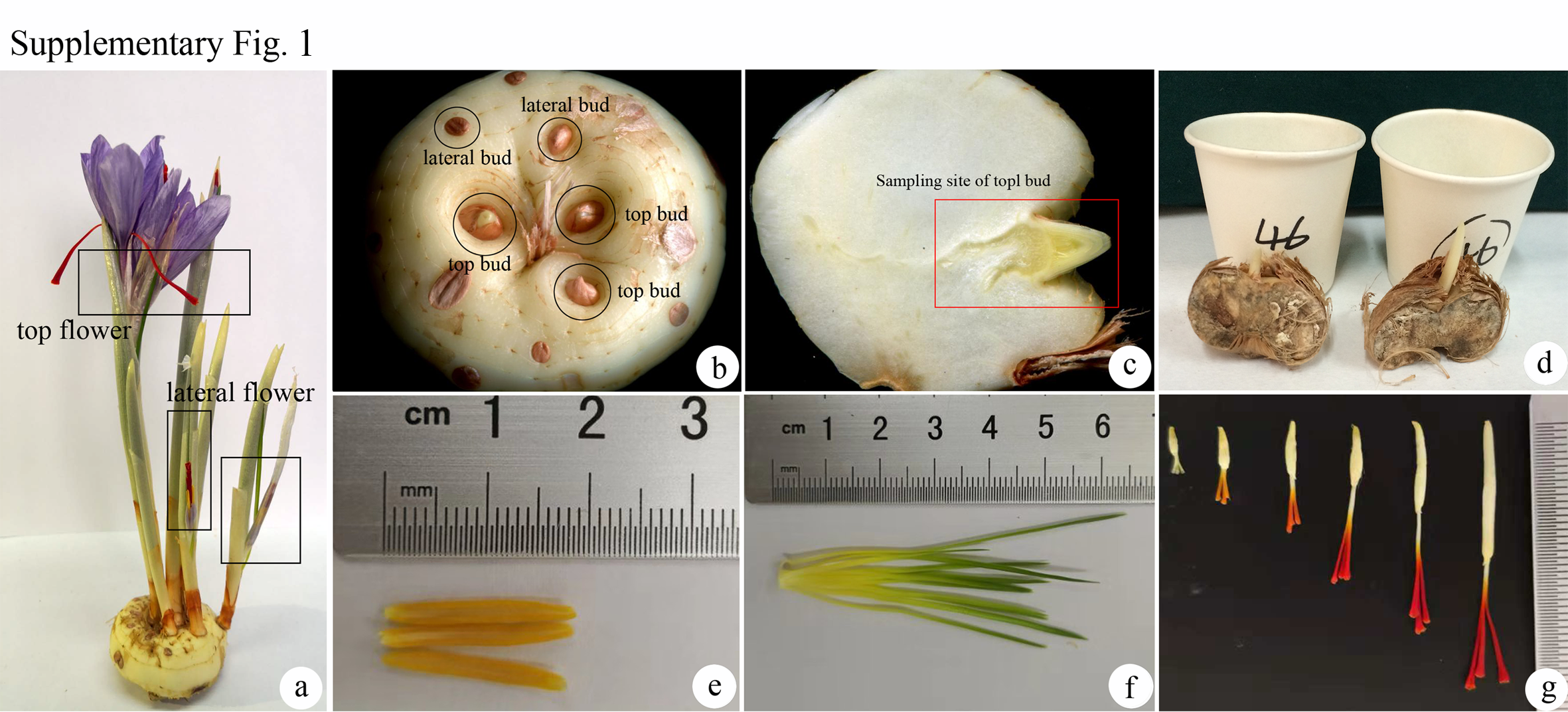

Supplement: Supplementary file 1 — Additional file 1: Figure S1. Morphology of the saffron samples; a: the whole saffron plant with flowers on both top and lateral buds. b: The sites of top buds and lateral buds of saffron crocus plants. c:Sampling sites of buds of saffron crocus. d: The flowering top bud (right) and paired cold-treated non-flowering top bud (left) of 20 g corms, which were spited into two parts and cultivated at room temperature or 10 °C separately. e: the pistils of the flower. f: the leaves of saffron crocus. g: the stamens of saffron crocus. [file 12864_2019_6200_MOESM1_ESM.tif]

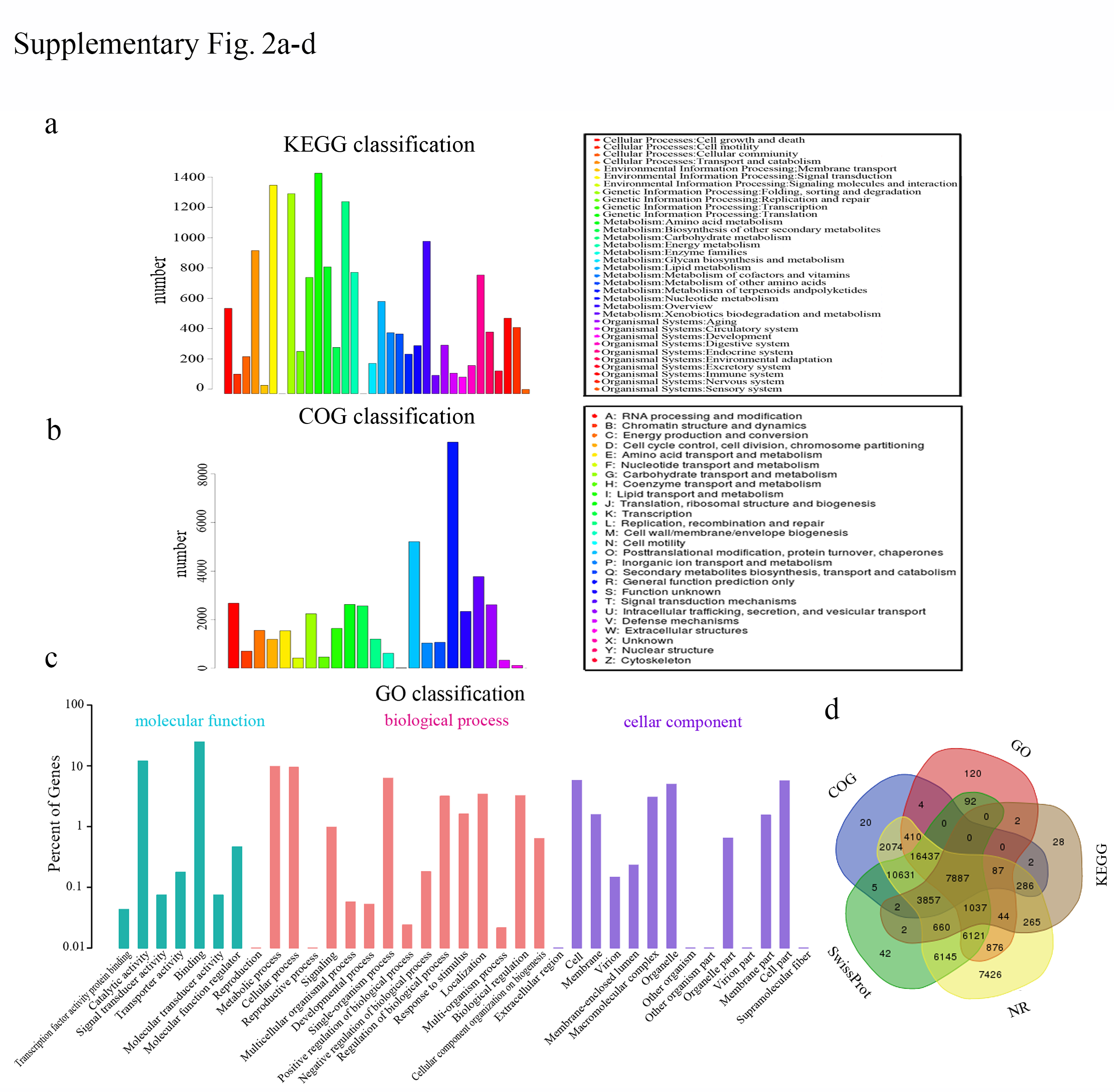

Supplement: Supplementary file 3 — Additional file 3: Figure S2. Functional annotations of 75,351 unigenes. a: KEGG pathway analysis of all unigenes. b: COG pathway analysis of all unigenes. c: GO pathway analysis of all unigenes. d: Venn diagram of functional annotations of NR, Swiss-Prot, KEGG, COG and GO databases. (TIF 19440 kb) [file 12864_2019_6200_MOESM3_ESM.tif]

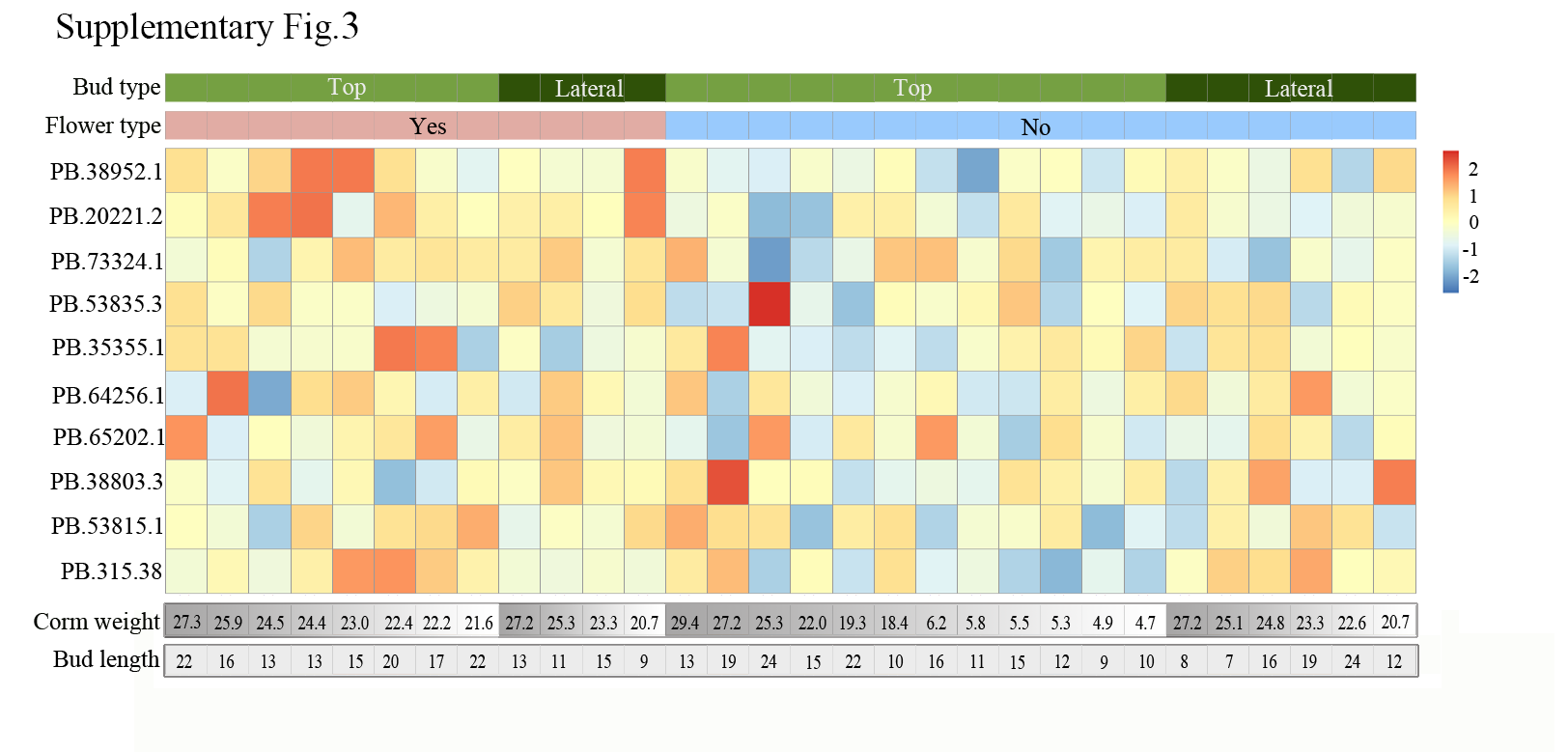

Supplement: Supplementary file 4 — Additional file 4: Figure S3. The heatmap shows the expression patterns of ten DEGs in 30 saffron crocus corms. [file 12864_2019_6200_MOESM4_ESM.tif]

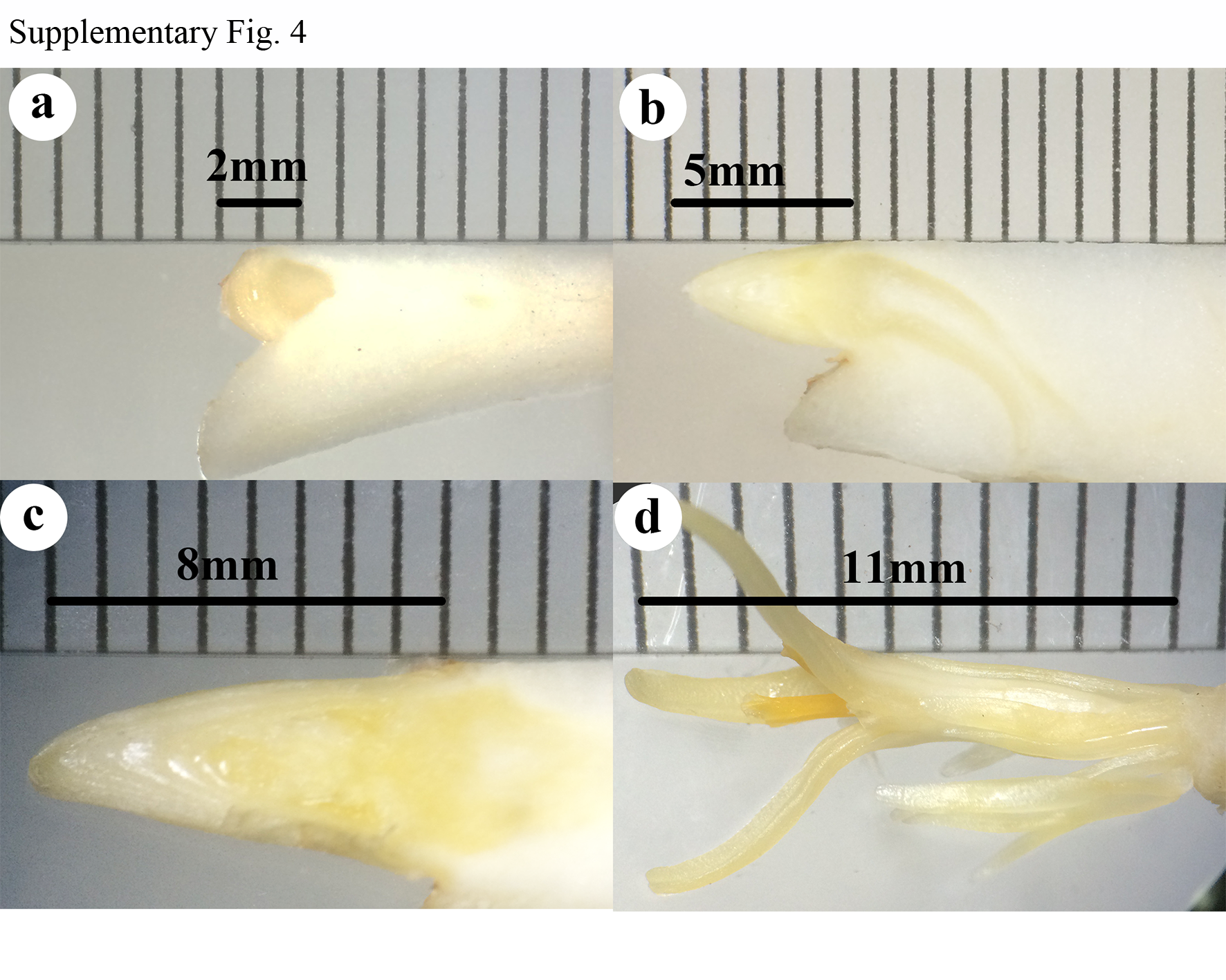

Supplement: Supplementary file 5 — Additional file 5: Figure S4. The top buds of saffron corms during the time-course from the dormant period to the start of the flowering season. a: resting bud; b: early stage of shoot growth; c: late stage of shoot growth; d: stage of visually distinguishable flower organ formation. [file 12864_2019_6200_MOESM5_ESM.tif]

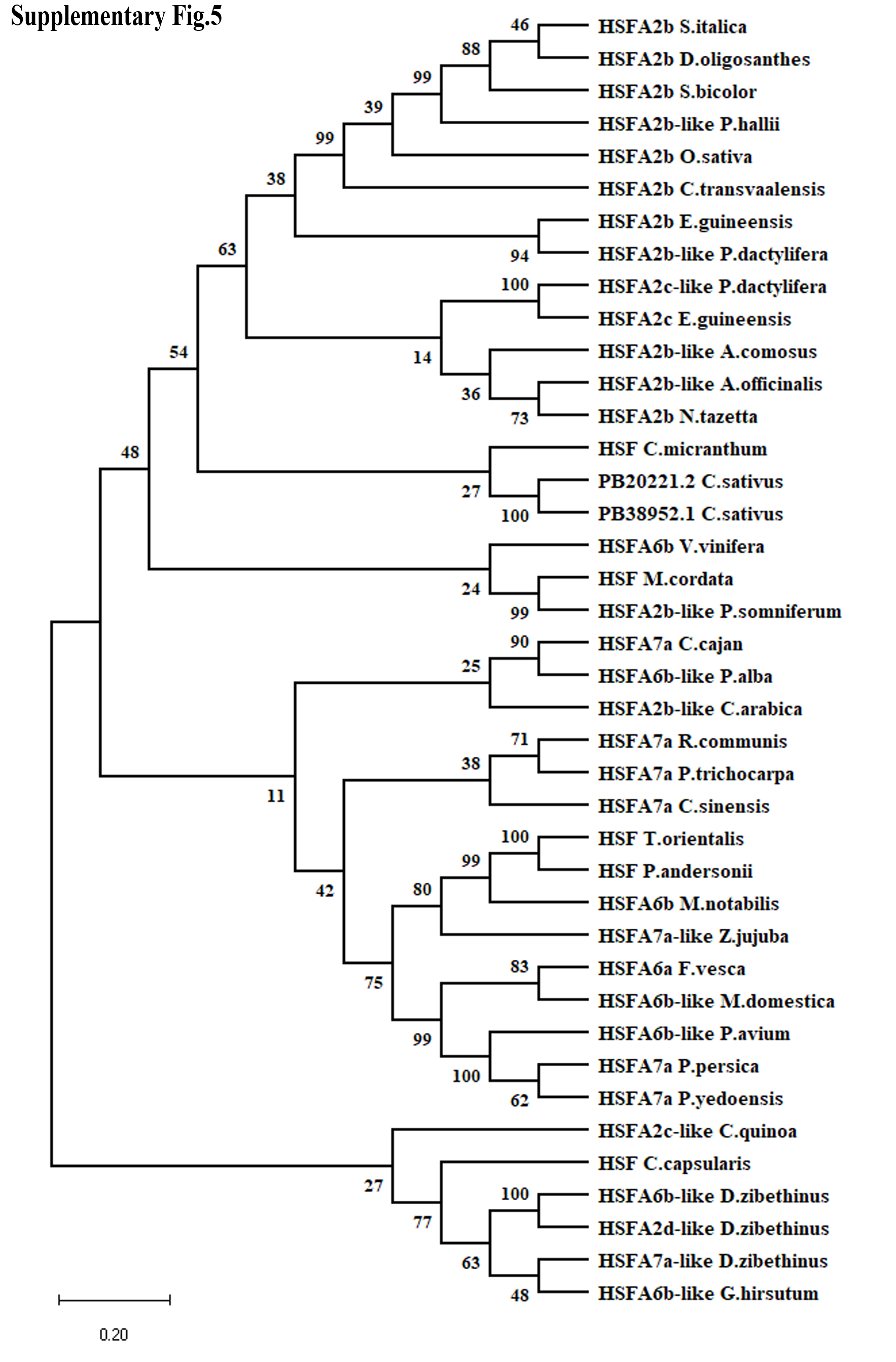

Supplement: Supplementary file 6 — Additional file 6: Figure S5. Phylogenetic analysis of a representative subset of 38 amino acid sequences belonging to the HSF family and the HSF-like deduced proteins isolated from saffron crocus (PB20221.2 and PB38952.1). Phylogenetic relationships of the sequences were examined using the neighbor-joining method. [file 12864_2019_6200_MOESM6_ESM.tif]
