# Supplementary material for: Single-molecule real-time transcript sequencing identified flowering regulatory genes in Crocus sativus
Source: BMC Genomics. 2019 Nov 14;20:857. doi: 10.1186/s12864-019-6200-5 (PMC6854690; doi:10.1186/s12864-019-6200-5)
Supplement: Supplementary file 2 — Additional file 2: Table S1. Primers of Unigenes for AS validation. Table S2. Primers of differentially expressed Unigenes for real-time qRT- PCR analysis. Table S3. PacBio Iso-seq data statistics. Table S4. Summary of reads of insert (ROIs). Table S5. Homology Analysis between mRNA sequence on NCBI and full-length unigene. Table S6. Sequencing data statistics of Illumina RNA-seq. Table S7. GO enrichment analysis of AS genes. Table S8. Specific AS events in flowering saffrons and non-flowering saffron crocus. Table S9. Number of DEGs in flowering and non-flowering saffron crocus. Table S10. DEGs only expressed in flowering top buds or non-flowering cold-treated top buds. Table S11. DEGs were only expressed in flowering top buds or non-flowering top buds of small corms. Table S12. DEGs were only expressed in flowering or non-flowering lateral buds. Table S13. Flower-related DEGs in three comparable experiments. [file 12864_2019_6200_MOESM2_ESM.zip › Table S9. Number of DEGs.doc]

| **Sample groups** | **Total DEGs** | **Down** | **Up** |
| --- | --- | --- | --- |
| flowering top buds vs matched non-flowering top buds with cold treatment | 1677 | 490 | 1187 |
| flowering top buds of 20 g vs non-flowering top buds of 6 g corms | 1086 | 199 | 887 |
| flowering-lateral buds vs matched non-flowering lateral buds | 267 | 223 | 44 |

**Table S9. Number of DEGs in flowering and non-flowering saffron crocus**
