# Supplementary material for: Single-molecule real-time transcript sequencing identified flowering regulatory genes in Crocus sativus
Source: BMC Genomics. 2019 Nov 14;20:857. doi: 10.1186/s12864-019-6200-5 (PMC6854690; doi:10.1186/s12864-019-6200-5)
Supplement: Supplementary file 2 — Additional file 2: Table S1. Primers of Unigenes for AS validation. Table S2. Primers of differentially expressed Unigenes for real-time qRT- PCR analysis. Table S3. PacBio Iso-seq data statistics. Table S4. Summary of reads of insert (ROIs). Table S5. Homology Analysis between mRNA sequence on NCBI and full-length unigene. Table S6. Sequencing data statistics of Illumina RNA-seq. Table S7. GO enrichment analysis of AS genes. Table S8. Specific AS events in flowering saffrons and non-flowering saffron crocus. Table S9. Number of DEGs in flowering and non-flowering saffron crocus. Table S10. DEGs only expressed in flowering top buds or non-flowering cold-treated top buds. Table S11. DEGs were only expressed in flowering top buds or non-flowering top buds of small corms. Table S12. DEGs were only expressed in flowering or non-flowering lateral buds. Table S13. Flower-related DEGs in three comparable experiments. [file 12864_2019_6200_MOESM2_ESM.zip › Table S2. Primers of DEGs for real-time qRT- PCR analysis.doc]

| **Unigene** | **Forward primer** | **Reverse primer** | **Product length** |
| --- | --- | --- | --- |
| *PB.20221.2* | GGCGTCACCTCCGTTGAAC | GGCGTCACCTCCGTTGAAC | 142bp |
| *PB.38952.1* | CGGAGTGGATGGAGAGATCG | CCCTGGAGCCTCTCTTCCAT | 128bp |
| *PB.73324.1* | TCCGAGGACGAGAGCAGAAC | AGGGTCGCAGTGTCCAGATT | 123bp |
| *PB.53835.3* | GCGGAGTCGAAGAGGAAAGC | CCCTCCGGATCACCATCTTC | 272bp |
| *PB.35355.1* | AAGTTGGTGGGTTAGTTC | ATCTGGCTTTGTTCTCAT | 153 bp |
| *PB.64256.1* | GGCGTTCGTCGTGTGGAG | GGCGGTGGATATCGCAGA | 194bp |
| *PB.65202.1* | ACTCTGACACGAAACACT | GTCCTTATCTTACCCTTG | 179bp |
| *PB.38803.3* | GTGGGAGTATGGCAGCAC | GTTTCATGGCGACTTGGT | 135bp |
| *PB.53815.1* | CGGCACGGTTCTGTGGAG | AGCTGGCCCTATTGGATCAG | 210bp |
| *PB.315.38* | GGTGGACCCACCGACAAGA | CCACCGCTCAGACCACAATT | 197bp |
| *tublin* | CGTGCGTTTGTTCACTGGTA | CCCACCTCTTCGTAATCCTTC | 104bp |

**Table S2. Primers of differentially expressed Unigenes for real-time qRT- PCR analysis**
