# Supplementary material for: Single-molecule real-time transcript sequencing identified flowering regulatory genes in Crocus sativus
Source: BMC Genomics. 2019 Nov 14;20:857. doi: 10.1186/s12864-019-6200-5 (PMC6854690; doi:10.1186/s12864-019-6200-5)
Supplement: Supplementary file 2 — Additional file 2: Table S1. Primers of Unigenes for AS validation. Table S2. Primers of differentially expressed Unigenes for real-time qRT- PCR analysis. Table S3. PacBio Iso-seq data statistics. Table S4. Summary of reads of insert (ROIs). Table S5. Homology Analysis between mRNA sequence on NCBI and full-length unigene. Table S6. Sequencing data statistics of Illumina RNA-seq. Table S7. GO enrichment analysis of AS genes. Table S8. Specific AS events in flowering saffrons and non-flowering saffron crocus. Table S9. Number of DEGs in flowering and non-flowering saffron crocus. Table S10. DEGs only expressed in flowering top buds or non-flowering cold-treated top buds. Table S11. DEGs were only expressed in flowering top buds or non-flowering top buds of small corms. Table S12. DEGs were only expressed in flowering or non-flowering lateral buds. Table S13. Flower-related DEGs in three comparable experiments. [file 12864_2019_6200_MOESM2_ESM.zip › Table S1. Primers of unigenes for AS validation.doc]

| Unigene | Forward primer | Reverse primer | |
| --- | --- | --- | --- |
| PB.174 | GCAGCCTCCTTTGCTCTCTC | | GGCCACAAGCATAATAATCCAAC |
| PB.144  PB.313  PB.988 | GCCCTAGCTGCCTTTCGTT  AGCTACCCATCCGACCTACACTGGCGATCGGGATCGATCT | | TCCAAGTCTCACAACAAAATTAT  GCGTCTTCAATGGATGGATCTT  CCAGGAAACATCCCTGGAAAC |

**Table S1 Primers of Unigenes for AS validation**
